# Concatenated 16S rRNA sequence analysis improves bacterial taxonomy

**DOI:** 10.12688/f1000research.128320.1

**Published:** 2022-12-19

**Authors:** Bobby Paul

**Affiliations:** 1 Department of Bioinformatics, Manipal School of Life Sciences, Manipal Academy of Higher Education, Manipal, Karnataka, 576104, India

**Keywords:** bacterial nomenclature, bacterial taxonomy, concatenated phylogeny, species-specific barcode reference library

## Abstract

**Background: **Microscopic, biochemical, molecular, and computer-based approaches are extensively used to identify and classify bacterial populations. Advances in DNA sequencing and bioinformatics workflows have facilitated sophisticated genome-based methods for microbial taxonomy although sequencing of the 16S rRNA gene is widely employed to identify and classify the bacterial community as a cost-effective and single-gene approach. However, the 16S rRNA sequence-based species identification accuracy is limited by multiple copies of the gene and their higher sequence identity between closely related species. The availability of a large volume of bacterial whole-genome data provided an opportunity to develop comprehensive species-specific 16S rRNA reference libraries.

**Methods:** The 16S rRNA copies were retrieved from the whole genomes in the complete stage at the Genome database. With defined rules, four 16S rRNA gene copy variants were concatenated to develop a species-specific reference library. The sequence similarity search was performed with a web-based BLAST program, and MEGA software was used to construct the phylogenetic tree.

**Results:** Using this approach, species-specific 16S rRNA gene libraries were developed for four closely related *Streptococcus* species ( *S. gordonii*, *S. mitis*, *S. oralis*, and *S. pneumoniae*). Sequence similarity and phylogenetic analysis using concatenated 16S rRNA copies yielded better resolution than single gene copy approaches.

**Conclusions:** The approach is very effective in classifying genetically related species and may reduce misclassification of bacterial species and genome assemblies.

## Introduction

The 16S ribosomal RNA (16S rRNA) encoding region is extensively studied to identify and classify bacterial species. The 16S rRNA is a conserved component of the 30S small subunit of a prokaryotic ribosome. The gene is ~1500 base pair (bp) long, and it consists of nine variable regions ( Reller *et al*. 2007; Sabat *et al*. 2017). For decades, the sequence of the 16S rRNA gene has been used as a potential molecular marker in culture-independent methods to identify and classify diverse bacterial communities ( Clarridge, 2004; Johnson *et al*. 2019). The 16S rRNA sequences are currently being used as an accurate and rapid method to study bacterial evolution, phylogenetic relationships, populations in an environment, and quantification of abundant taxa ( Vetrovsky and Baldrian, 2013; Srinivasan *et al*. 2015; Peker *et al*. 2019).

Despite the wide range of applications, a few shortcomings limit the accuracy of results derived through the 16S rRNA sequence analysis. One such aspect is that the 16S rRNA gene has poor discriminatory power at the species level ( Winand *et al*. 2020), and the copy number can vary from 1 to 15 or even more ( Vetrovsky and Baldrian, 2013; Winand *et al*. 2020). The presence of multiple variable copies of this gene makes distinct data for a species. Hence, gene copy normalization (GCN) is necessary prior to sequence analysis. However, studies show that the GCN approach does not improve the 16S rRNA sequence analyses in real scenarios and suggests a comprehensive species-specific catalogue of gene copies ( Starke *et al*. 2021). Secondly, the intra-genomic variations between the 16S rRNA gene copies were observed in several bacterial genome assemblies ( Paul *et al*. 2019). Only a minority of the bacterial genomes harbor identical 16S rRNA gene copies, and sequence diversity increases with increasing copy numbers ( Vetrovsky and Baldrian, 2013). Further, currently available 16S rRNA-based bioinformatics approaches are not always amenable to classify bacterium at the species level due to high inter-species sequence similarities ( Peker *et al*. 2019; Deurenberg *et al*. 2017).

A few other issues are also related to the sequencing and bioinformatics analysis of 16S rRNA gene regions. These include the purity of bacterial isolates, the quality of isolated DNA, and the possibility of chimeric molecules ( Janda and Abbott, 2007; Church *et al*. 2020). Base-call errors can also mislead the sequence identity and phylogenetic inferences ( Alachiotis *et al*. 2013). The other concerns on sequence-based analysis, comparison, and species identification include the number of base ambiguities processed, gaps generated during sequence comparison, and algorithm (local or global) used for the sequence alignment. The local alignment algorithm is extensively used for sequence similarity-based species identification. Several studies were conducted to identify the best variable region or combination of variable regions for bacterial classification, and a consensus remains to be implemented ( Janda and Abbott, 2007; Johnson *et al*. 2019; Winand *et al*. 2020). Usage of misclassified sequence as a reference and improper bioinformatics workflows mislead the bacterial taxonomy. Further, the growth of bioinformatics and genetic data has placed genome-based microbial classification with researchers with little or no taxonomic experience, which may also mislead the bacterial taxonomy ( Baltrus, 2016).

A few bacterial identification systems with high resolution have been developed using the sequence of polymerase chain reaction (PCR) amplified ∼4.5 kb long 16S–23S rRNA regions (Benítez-Páez and Sanz, 2017; Sabat *et al*. 2017; Kerkhof *et al*. 2017). However, these approaches have a few limitations, such as the lack of reference 16S–23S rRNA sequence databases and complementary bioinformatics resources for reliable species identification ( Sabat *et al*. 2017). The recent advancements in bioinformatics workflows ( Winand *et al*. 2020; Schloss, 2020) and reference databases such as SILVA, EzBioCloud ( Quast *et al*. 2013; Yoon, 2017) improved 16S rRNA-based bacterial taxonomy. However, a few recent genome-based studies highlighted the misclassification incidences in bacterial species and genome assemblies ( Steven *et al*. 2017; Martínez-Romero, *et al*. 2018; Mateo-Estrada *et al*. 2019; Bagheri *et al*. 2020).

Nowadays, conventional and high throughput sequencers can amplify all the nine variable regions of the 16S rRNA gene. Although, many 16S rRNA-based bacterial identification studies lack a complete set of variable regions ( Stackebrandt *et al*. 2021). The classical and high throughput sequencing technologies produce a large volume of whole-genome data. There is an urgent need to translate the genomic data for convenient microbiome analyses that ensure clinical practitioners can readily understand and quickly implement it ( Church *et al*. 2020). Hence, the study intended to demonstrate a workflow to develop species-specific concatenated 16S rRNA reference libraries and its analysis. The species-specific libraries can yield better resolution in sequence similarity and phylogeny based bacterial classification approaches.

## Methods

### Estimation of variations in intra-genomic 16S rRNA gene copies

Sequence alignment of 16S rRNA copies at the intra-genomic level shows a higher degree of variability in species belonging to the *Firmicutes* and *Proteobacteria* ( Vetrovsky and Baldrian, 2013; Ibal *et al*. 2019). Hence, the study used eight 16S rRNA copies (Underlying data: Supplementary data 1 ( Paul, 2022)) retrieved from the whole genome of *Enterobacter asburiae* strain ATCC 35953 (NZ_CP011863.1). The BLAST+ 2.13.0 (RRID:SCR_004870; Altschul *et al*. 1990) and Clustal Omega 1.2.4 (RRID:SCR_001591; Sievers *et al*. 2011) sequence alignment algorithms were used to estimate intra-genomic variability between the 16S rRNA gene copies. Phylogenetic relatedness between intra-genomic 16S rRNA copies were estimated using the Maximum Likelihood method (Tamura-Nei model; 500 bootstrap replicates) with MEGA software (version 11; RRID: SCR_000667; Kumar *et al*. 2018).

### Construction of species-specific concatenated 16S rRNA reference libraries

Previous studies have reported that several bacterial species share more than 99% sequence identity in the 16S rRNA encoding region. Hence, the 16S rRNA-based bacterial identification methods failed to discriminate such genetically related species ( Deurenberg *et al*. 2017; Devanga-Ragupathi *et al*. 2018). It has been reported that *Streptococcus mitis* and *Streptococcus pneumoniae* are almost indistinguishable from each other based on the sequence similarity of their 16S rRNA regions ( Reller *et al*. 2007; Lal *et al*. 2011). To develop species-specific barcode reference libraries, the study used 16S rRNA gene copies from whole-genome assemblies of four closely related species of *Streptococcus* ( *S. gordonii*, *S. mitis*, *S. oralis,* and *S. pneumoniae*).

More than 463,000 whole-genome assemblies are currently available for prokaryotes at the Genome database (RRID:SCR_002474; https://www.ncbi.nlm.nih.gov/genome). Most microbial genomes were sequenced with high throughput sequencing technologies such as Illumina/Ion-Torrent (short read sequencing) and PacBio/Nanopre (long read sequencing). Further, many of these whole-genome assemblies are derived through a hybrid assembly of short and long read sequence data. The large volume of high throughput data can be effectively used to develop advanced genome-based approaches for microbial systematics. The genomic data is available in four assembly completion levels (contig, scaffold, chromosome, and complete). However, the study used only the genomes assemblies in the 'complete' stage to retrieve 16S rRNA gene copies.

The study retrieved full-length 16S rRNA gene copies from 16 genome assemblies belonging to four *Streptococcus* species ( *S. gordonii*, *S. mitis*, *S. oralis,* and *S. pneumoniae*). The detailed information on the dataset used to develop species-specific concatenated reference libraries is provided in Table 1 and the sequences are provided in the underlying data (Supplementary data 2 ( Paul, 2022)). To maintain equal length, sequences were trimmed out beyond the universal primer pair fD1-5'-GAG TTT GAT CCT GGC TCA-3' and rP2-5'-ACG GCT AAC TTG TTA CGA CT-3' ( Weisburg *et al*. 1991) for full-length 16S rDNA amplification. The study used MEGA 11 software to perform multiple sequence alignment and identify the intra-species parsimony informative (Parsim-info) variable sites. A species-specific barcode reference library covering entire Parsim-info variable sites was constructed by concatenating four 16S rRNA gene copies representing four different strains of a species. The rationale behind the selection of four copies for a species-specific barcode reference library is: (i) a maximum of four variations can be found on a single site, and (ii) earlier studies have shown that the mean 16S rRNA copies per genome is four ( Vetrovsky and Baldrian, 2013).

**Table 1.** Details of whole genome assemblies used for the development of concatenated 16S rRNA reference libraries. One copy of 16S rRNA gene from each strain is used for the concatenation.

Species	Strains	Genome accession number	No. of 16S rRNA gene copies	Sequencing platform	Species-specific library name	Library length (bp)	No. of Parsim-info sites
*S. gordonii*	FDAARGOS 1454	CP077224.1	4	PacBio; Illumina	*S.gordonii-*Ref-I	6076	7
	NCTC7868	LR134291.1	4	PacBio			
	KCOM 1506	CP012648.1	5	Illumina			
	NCTC9124	LR594041.1	4	PacBio			
*S. mitis*	B6	NC_013853.1	4	NA	*S.mitis-*Ref-I	6033	11
	KCOM 1350	CP012646.1	3	Illumina			
	SVGS 061	CP014326.1	4	PacBio; Illumina			
	NCTC 12261	CP028414.1	4	PacBio			
*S. oralis*	NCTC 11427	LR134336.1	4	PacBio	*S.oralis-*Ref-I	6038	24
	34	CP079724.1	4	Illumina; Nanopore			
	FDAARGOS 886	CP065706.1	4	PacBio; Illumina			
	F0392	CP034442.1	4	PacBio			
*S. pneumoniae*	475	CP046355.1	4	PacBio	*S.pneumoniae*-Ref-I	6032	6
	NU83127	AP018936.1	4	Nanopore; Illumina			
	NCTC7465	LN831051.1	4	PacBio			
	6A-10	CP053210.1	4	PacBio			

### Demonstration of concatenated 16S rRNA in sequence similarity and phylogeny

The study analyzed a few cases to demonstrate the classical sequence similarity and phylogenetic analysis using concatenated species-specific 16S rRNA reference libraries. The study used nine Sanger sequenced 16S rRNA gene copies showing higher sequence similarity with multiple species of *Streptococcus* retrieved from the GenBank database (RRID:SCR_002760). The web based BLAST2 (version 2.13.0) program for aligning two or more sequences was used to estimate the maximum score, total alignment score, and sequence identity. A single copy of the 16S rRNA region derived through Sanger sequencing or retrieved from a whole-genome assembly can be considered as ‘Query sequence’. The concatenated species-specific reference libraries must be provided in the ‘Subject sequence’ section. To perform phylogenetic analysis, it is mandatory that the target sequence (length = n bp) has to be concatenated four times (length = 4 × n bp), appending next to the last base. Phylogenetic relatedness was estimated using the Maximum Likelihood method (Tamura-Nei model; 500 bootstrap replicates) with MEGA 11 software.

## Results

### Intra-genomic 16S rRNA variations in *Enterobacter asburiae*

**Figure 1.**
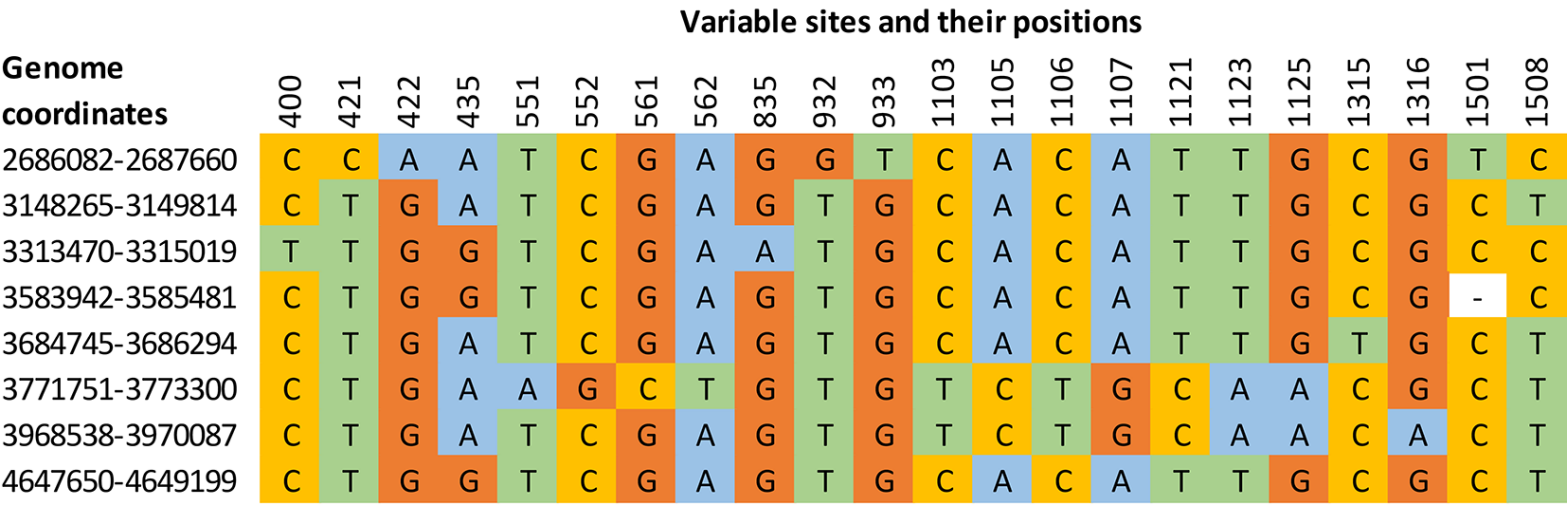
Multiple sequence alignment of eight intra genomic 16S rRNA gene copies from *Enterobacter asburiae* strain ATCC 35953 (NZ_CP011863.1) showing 22 variable sites.

Historically, the 16S rRNA gene sequences were used to identify known and new bacterial species. However, this method is impacted by several factors such as amplification efficiency, poor discriminatory power at the species level, multiple polymorphic 16S rRNA gene copies, and improper bioinformatics workflows for the data analysis. The *E. asburiae* genome had eight 16S rRNA gene copies that showed a mean identity of 99.29% in sequence alignment using Clustal Omega (global alignment), whereas BLAST (local alignment) analysis resulted in an average of 99% identity between the copies ( Table 2). Hence, the selection of an appropriate algorithm has a significant role in the estimation of percent identity, and a vital role in sequence-based species delineation. Global sequence alignment programs generally perform better for highly identical sequence pairs, and the algorithm considers all the bases for the estimation of sequence identity. The multiple sequence alignment showed 22 variable sites in 16S rRNA gene copies of the *E. asburiae* genome ( Figure 1).

**Table 2.** Percent identity of eight intra genomic 16S rRNA regions from *Enterobacter asburiae* strain ATCC 35953 (NZ_CP011863.1). Percent identity given below the diagonal line is calculated with Clustal Omega software (Mean identity: 99.29%) and those above the diagonal line were calculated with the BLASTN program (Mean identity: 99.00%). Genome coordinates of 16S rRNA copies: R1: 2686082–2687660 (1579 bp); R2: 3148265–3149814 (1550 bp); R3: 3313470–3315019 (1550 bp); R4: 3583942–3585481 (1540 bp); R5:3684745–3686294 (1550 bp); R6: 3771751–3773300 (1550 bp); R7: 3968538–3970087 (1550 bp); R8: 4647650–4649199 (1550 bp)

16S rRNA copies	R1	R2	R3	R4	R5	R6	R7	R8
**R1**		98.10	98.04	97.47	98.04	97.47	97.59	98.04
**R2**	99.10		99.74	99.23	99.94	99.29	99.48	99.94
**R3**	98.97	99.74		99.23	99.68	99.03	99.23	99.81
**R4**	98.90	99.41	99.41		99.16	98.52	98.71	99.29
**R5**	99.03	99.94	99.68	99.35		99.23	99.42	99.87
**R6**	98.39	99.29	99.03	98.70	99.23		99.68	99.23
**R7**	98.58	99.48	99.23	98.89	99.42	99.68		99.42
**R8**	99.03	99.94	99.81	99.48	99.87	99.23	99.42	

**Figure 2.**
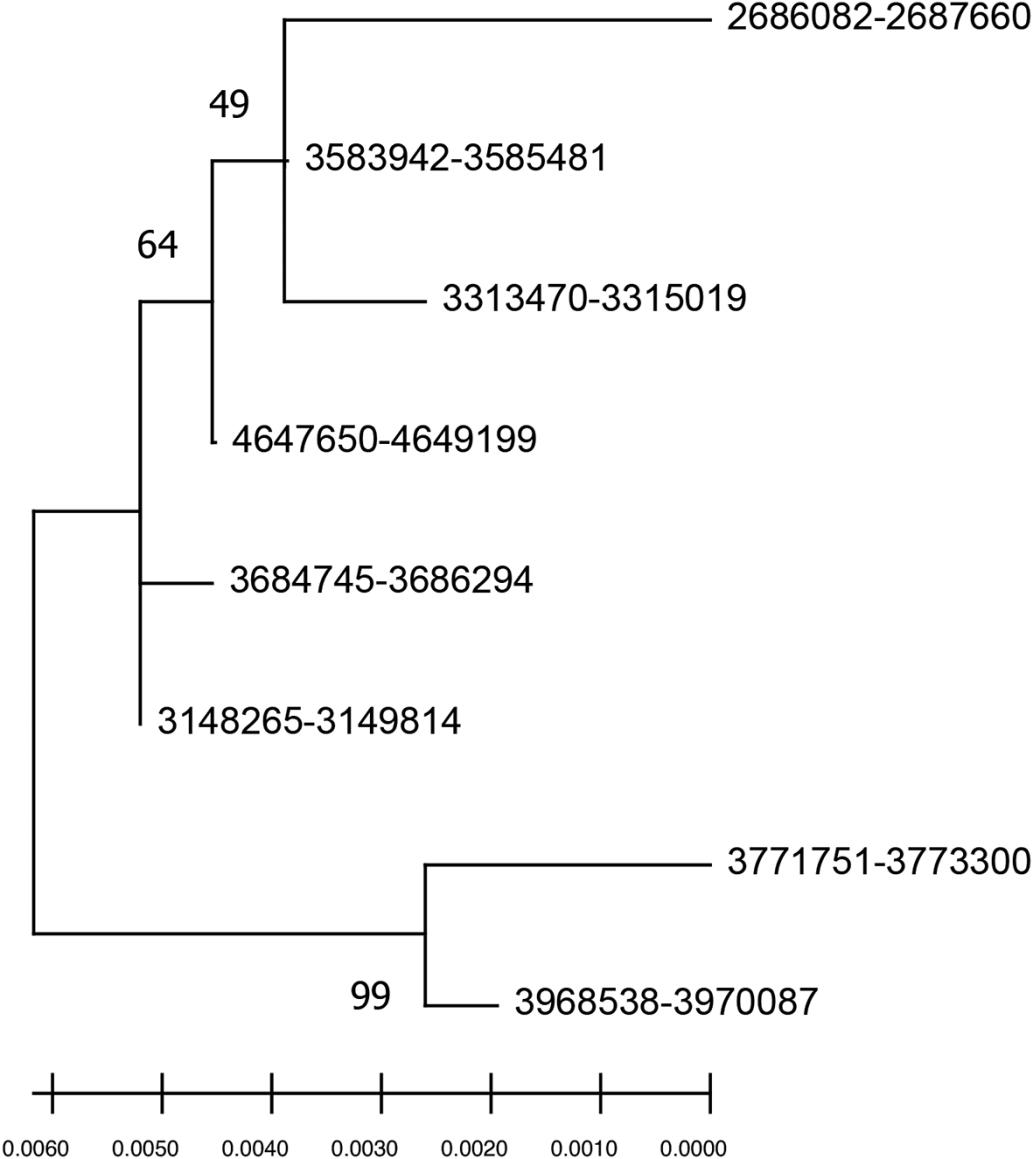
Phylogenetic tree of eight intra genomic 16S rRNA gene copies from *Enterobacter asburiae* strain ATCC 35953 (NZ_CP011863.1). The node label denotes the coordinate of 16S rRNA regions in the genome.

The evolutionary relationship between species is usually represented in a phylogenetic tree drawn using a single barcode gene, multiple genes, or whole genomes. However, bacterial species nomenclature is mainly designated based on the confidence obtained from the phylogenetic tree derived through single copy 16S rRNA analysis. To highlight how the intra-genomic 16S rRNA variations influence the species delineation, a phylogenetic tree was constructed using eight 16S rRNA gene copies of *E. asburiae* reference genome showing multiple nodes ( Figure 2). The sequence similarity and phylogeny-based analysis indicate that the intra-genomic variations in 16S rRNA copies may mislead the bacterial taxonomy in single gene copy approaches.

### Species-specific concatenated 16S rRNA libraries

The study selected four *Streptococcus* species ( *S. gordonii*, *S. mitis*, *S. oralis*, and *S. pneumoniae*) to construct species-specific concatenated 16S rRNA reference libraries. The study used 16S rRNA copies retrieved from four whole genome assemblies in the ‘complete’ stage to construct a species-specific barcode library. Four copies of the 16S rRNA gene are required to construct the concatenated library for a species. The details of constructed species-specific libraries are listed in Table 1 and the sequence is provided in the underlying data (Supplementary data 3 ( Paul, 2022)). The 16S rRNA sequence analysis shows 24 Parsim-info variable sites for *S. oralis*, 11 variations in *S. mitis*, seven variations in *S. gordonii,* and six variations found in *S. pneumoniae.* The observed intra-species Parsim-info variable sites are residing on both conserved and variable regions of the 16S rRNA gene.

The study used full-length 16S rRNA copies from four different strains to highlight the variations at the species level. However, a large volume of partial 16S rRNA sequences are available in the public genetic databases. In such cases, a species-specific concatenated 16S rRNA reference library can be developed with partial sequences. Intra-species variation on 16S rRNA gene copies influences the sequence based bacterial taxonomy. Hence, the concatenated 16S rRNA approach yields better resolution than single copy analysis in classical sequence similarity and phylogeny based species identification approaches.

### Demonstration of concatenated 16S rRNA based species identification

**Figure 3.**
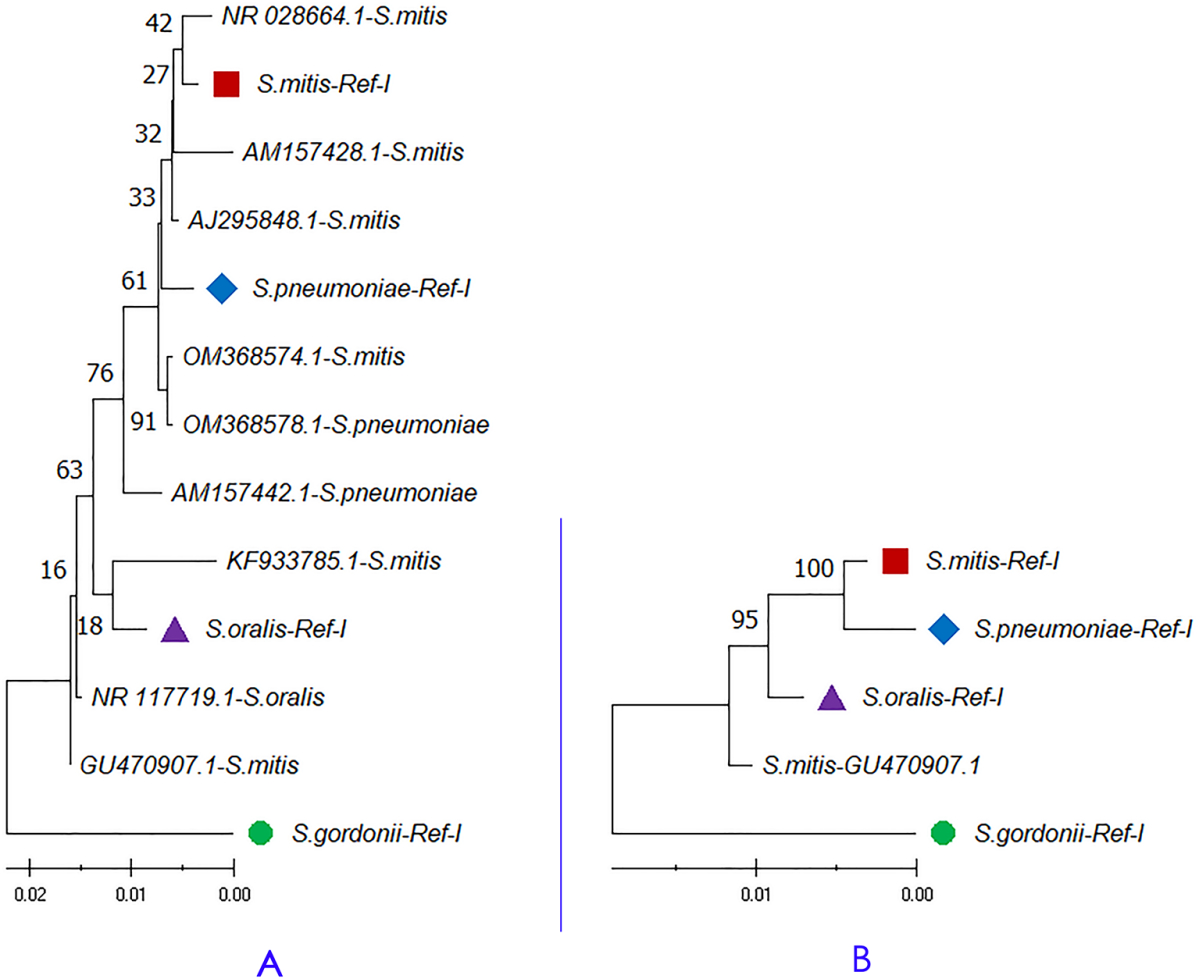
A) Phylogenetic analysis of randomly selected 16S rRNA sequences classified as *Streptococcus* species. B) Concatenated 16S rRNA phylogeny of *Streptococcus mitis* sequence (Accession Number GU470907.1) showed 100% identity with *Streptococcus oralis* genome (Accession Number CP034442.1) in a BLAST based sequence similarity search. The node name highlighted in shapes (●, ■, ▲, ◆) represents the four species-specific reference libraries.

The study compared nine 16S rRNA sequences representing *Streptococcus* species ( Table 3) with species-specific concatenated reference libraries. Concatenated sequence analysis gives better resolution in sequence similarity search and phylogenetic analysis. The sequence accession numbers GU470907.1 and KF933785.1 classified as *S. mitis* showed a higher maximum and total alignment score with *S. oralis* than *S. mitis* ( Table 3). Whereas the sequence (OM368574.1; classified as *S. mitis*) showed a higher sequence alignment score with *S. pneumoniae.* Figure 3A shows a maximum likelihood tree of the nine 16S rRNA gene sequences with four concatenated species-specific reference libraries. The concatenated GU470907.1 and KF933785.1 sequences showed a phylogenetic relationship with *S. oralis* and sequence OM368574.1 was genetically related to *S. pneumoniae.* These results indicate that the species-specific concatenated 16S rRNA reference libraries have great potential in the taxonomic classification. Hence, the study suggests the usage of concatenated variable 16S rRNA copies for sequence similarity and phylogeny-based species identification. A species-specific reference library with concatenated 16S rRNA gene copies provides better resolution in phylogenetic analysis than the single copy inference.

**Table 3.** Similarity of selected sequences against the concatenated species-specific 16S rRNA reference libraries.

GenBank Accession Number	Species	*S. gordonii*-Ref-I			*S. mitis*-Ref-I			*S. oralis*-Ref-I			*S. pneumoniae*-Ref-I		
		Max Score	Total Score	Identity (%)	Max Score	Total Score	Identity (%)	Max Score	Total Score	Identity (%)	Max Score	Total Score	Identity (%)
AJ295848.1	*S. mitis*	2495	9967	96.45	2769	11027	99.80	2758	10851	99.67	2752	10982	99.60
AM157428.1	*S. mitis*	2462	9845	96.05	2724	10866	99.27	2702	10685	99.01	2708	10805	99.07
NR_028664.1	*S. mitis*	2499	9991	96.45	2776	10979	99.87	2750	10864	99.54	2724	10888	99.27
GU470907.1	*S. mitis*	2536	10096	96.91	2715	10796	99.14	2787	10936	100	2091	10716	98.87
KF933785.1	*S. mitis*	2466	9832	96.06	2667	10593	98.54	2673	10650	98.61	2632	10502	98.15
OM368574.1	*S. mitis*	2475	9896	96.24	2754	10968	99.67	2732	10814	99.40	2760	10990	99.73
OM368578.1	*S. pneumoniae*	2475	9896	96.24	2754	10968	99.67	2732	10814	99.40	2760	10990	99.73
AM157442.1	*S. pneumoniae*	2470	9863	96.12	2702	10779	99.01	2715	10726	99.14	2702	10777	99.01
NR_117719.1	*S. oralis*	2531	10074	96.84	2710	10774	99.07	2787	10925	100	2697	10739	98.94

## Discussion

Sequencing and analysis of the 16S rRNA encoding region is considered a conventional and robust method for identifying and classifying the bacterial species. The barcode gene is widely used in sequence similarity, phylogeny, and metagenome-based species identification. However, the accuracy of bacterial taxonomy based on 16S rRNA barcode regions is limited by the intra-genomic heterogeneity of multiple 16S rRNA gene copies and significant sequence identity of this gene between closely related taxa. Further, discrimination of closely related species identification through sequences of the 16S rRNA gene is a challenge, and it may lead to species misidentification ( Boudewijns *et al*. 2006; Church *et al*. 2020). About 15% of the genomes have only a single copy of the 16S rRNA gene, and only a minority of bacterial genomes harbour identical 16S rRNA gene copies ( Vetrovsky and Baldrian, 2013). The 16S rRNA gene copies can vary from 1 to 15 in a genome, and the copy number of variations is taxon specific ( Vetrovsky and Baldrian, 2013). Sequence diversity increases with the increasing 16S rRNA copy numbers. The 16S rRNA sequence variation can even be found at intra-genomic level or in different strains of a species as well. Amplification of limited number of variable regions cannot achieve the taxonomic resolution achieved by sequencing the entire gene ( Johnson *et al*. 2019). Usage of misclassified 16S rRNA sequences as a reference and inappropriate bioinformatics workflows also mislead the taxonomic assignment. To overcome these challenges, it is important to translate high throughput microbial genomic data into meaningful, actionable information that clinicians can readily understand and quickly implement for bacterial identification. Hence, the study intended to develop a species-specific catalogue of concatenated 16S rRNA gene copies that can yield better inference in sequence similarity and phylogenetic analysis.

Several bioinformatics resources are extensively used for the 16S rRNA sequence analysis and bacterial identification. However, several researchers report the sequence similarity derived through a local alignment algorithm. Earlier reports have suggested that the species belonging to the taxa Gammaproteobacteria show higher intra-species variability ( Vetrovsky and Baldrian, 2013). Hence, the study estimated the percent identity of intra-genomic 16S rRNA gene copies of *Enterobacter asburiae* using local and global alignment algorithms. The reference genome of *E. asburiae* has eight 16S rRNA gene copies in its genome. The BLAST and Clustal sequence alignment algorithms yielded marginally varying results for the intra-genomic 16S rRNA gene copies. Local alignment algorithms may not consider base mismatches at the sequence ends for calculating percent identity, while global alignment algorithms consider entire bases. Therefore, global sequence alignment is best for estimating intra and inter-species identity for single gene copies. However, BLAST can calculate the total alignment score with multiple paralogue regions. Hence, web-based BLAST2 is suggested for estimating the sequence similarity using concatenated barcode reference libraries.

The GenBank ( Leray *et al*. 2019) and NCBI 16S RefSeq database for bacteria ( Winand *et al*. 2020) are reliable for species-level identification and classification. However, few earlier studies have highlighted the misclassification of species and genome assemblies in public genetic databases ( Parks *et al*. 2018; Varghese *et al*. 2015). For example, the 16S rRNA sequence accession number (Ac. No.) LT707617.1 shows the organism as *Streptococcus mitis.* Conventional BLAST-based sequence similarity search shows the highest identity of 99.60% with *S. mitis* 16S rRNA sequence (Ac. No. AB002520.1). However, the 16S rRNA sequence (Ac. No. LT707617.1) did not show significant similarity with other 16S rRNA reference sequences available for *S. mitis.* Further, the sequence also shows 99.44% identity with reference 16S rRNA sequences of *S. gordonii.* Hence, the study performed a sequence alignment of the sequence (Acc. No. LT707617.1) against species-specific concatenated 16S rRNA reference libraries for *S. gordonii* ( *S.gordonii-*Ref-I), and *S. mitis* ( *S.mitis-*Ref-I). The alignment resulted in a significant identity of 99.44% with *S.gordonii-*Ref-I (2279 maximum and 9041 total alignment score) than *S.mitis*-Ref-I (97.13% identity with 2119 maximum and 8449 total alignment score). Single copy BLAST results may show only a minor fraction of the difference in percent identity and maximum or total alignment score for closely related species. However, sequence similarity estimation using species-specific concatenated reference libraries shows marginal difference in total alignment score, as it is aligned against four copies. Hence, 16S rRNA analysis with a species-specific concatenated barcode reference library will give better accuracy for bacterial classification than approaches using a single copy.

Several 16S rRNA sequences show 100% identity with multiple species, which is the major challenge in sequence-based species identification. For example, the 16S rRNA sequence from *S. mitis* (Ac. No. GU470907.1; 1522 bp) shares 100% identity with the 16S rRNA gene from *S. oralis* strain ATCC 35037 genome (Ac. No. CP034442.1). Hence, the sequence (GU470907.1) aligned against the species-specific concatenated reference libraries for *S. oralis* ( *S.oralis-*Ref-I), and *S. mitis* ( *S.mitis-*Ref-I). The result showed 100% identity with *S. oralis* (2787 maximum and 10936 total alignment score), and 99.14% identity with *S. mitis* (2715 maximum and 10796 total alignment score). Further, a phylogenetic tree of GU470907.1 (1509 × 4 = 6036 bp) with reference libraries *S.mitis-*Ref-I, and *S.oralis-*Ref-I was plotted. The maximum likelihood-based phylogenetic tree showed that the *S. mitis* (GU470907.1) sequence is more closely related to *S. oralis* than *S. mitis* ( Figure 3B). Concatenated 16S rRNA-based estimation of sequence similarity and a phylogenetic inference provides better resolution than single-gene approaches. These results show that concatenated 16S rRNA approach is very effective in discriminating even genetically related bacterial species. Further, other studies also highlighted that the phylogenetic tree inferred from vertically inherited protein sequence concatenation provided higher resolution than those obtained from a single copy ( Ciccarelli *et al*. 2006; Thiergart *et al*. 2014).

Recent phylogenetic studies using concatenated multi-gene sequence data highlighted the importance of incorporating variation in gene histories, which will improve the traditional phylogenetic inferences ( Devulder *et al*. 2005; Johnston *et al*. 2019). Further, one type of analysis should not be relied upon, instead, and to a certain extent, integrated bioinformatics approaches can avoid misclassification. As a cost-effective approach, the study combined substantial variations in 16S rRNA gene copies from a species to examine the performance of the single gene concatenation approach. Analyses using a concatenated 16S rRNA gene approach have some advantages: (i) the gene is present in all the bacterial species, (ii) the gene is weakly affected by horizontal gene transfer, (iii) the approach is very cost-effective, (iv) there is a large volume of reference genomic data available for several bacterial species, (v) it is effective in discriminating closely related bacterial species, (vi) the analyses can be performed in a computer with minimum configuration, and (vii) the analyses can be employed with available tools for sequence similarity and molecular phylogeny.

## Conclusions

The concatenated 16S rRNA analyses drew the following suggestions:

• Full-length 16S rRNA gene amplification provides better accuracy than inference from a partial gene with a limited number of variable regions.
• Prior to the analysis, trim the bases beyond the primer ends and correct the base-call errors, which will avoid several mismatches in the sequence alignment.
• Estimation of mean 16S rRNA identity at the intra-species level helps to classify the species having a higher degree of intra-genomic 16S rRNA heterogeneity.
• Use full-length 16S rRNA gene copies from whole-genome assemblies (in 'complete' stage) rather than partial sequences available from the public genetic databases to construct species-specific concatenated 16S rRNA libraries and further downstream analysis.
• Distinct four 16S rRNA gene copies cover all the Parsim-Info variable sites and can be used to construct a concatenated species-specific reference library.
• The total alignment score can be considered if the query sequence shows more or less the same percent identity with multiple species.
• Do not rely only on sequence similarity; make a final decision based on the phylogenetic inference.

## Data Availability

Zenodo: Underlying data for ‘Concatenated 16S rRNA sequence analysis improves bacterial taxonomy’. https://www.doi.org/10.5281/zenodo.7384708 ( Paul, 2022) This project contains the following underlying data:
• Supplementary data 1: The 16S rRNA copies retrieved from the whole genome of *Enterobacter asburiae* strain ATCC 35953. • Supplementary data 2: Full-length 16S rRNA gene copies retrieved from 16 genome assemblies belonging to four *Streptococcus* species ( *S. gordonii*, *S. mitis*, *S. oralis*, and *S. pneumoniae*). • Supplementary data 3: Species-specific concatenated 16S rRNA libraries constructed for four *Streptococcus* species ( *S. gordonii*, *S. mitis*, *S. oralis*, and *S. pneumoniae*). Data are available under the terms of the Creative Commons Attribution 4.0 International license (CC-BY 4.0) GenBank: *Streptococcus gordonii* strain FDAARGOS 1454 chromosome, complete genome. Accession number CP077224.1. https://www.ncbi.nlm.nih.gov/nuccore/CP077224.1 GenBank: *Streptococcus gordonii* strain NCTC7869, chromosome 1, complete genome. Accession number LR134291.1. https://www.ncbi.nlm.nih.gov/nuccore/LR134291.1 GenBank: *Streptococcus gordonii* strain KCOM 1506 (=ChDC B679), complete genome. Accession number CP012648.1. https://www.ncbi.nlm.nih.gov/nuccore/CP012648.1 GenBank: *Streptococcus gordonii* strain NCTC9124, chromosome 1, complete genome. Accession number LR594041.1. https://www.ncbi.nlm.nih.gov/nuccore/LR594041.1 GenBank: *Streptococcus mitis* B6, complete genome. Accession number NC_013853.1. https://www.ncbi.nlm.nih.gov/nuccore/NC_013853.1 GenBank: *Streptococcus mitis* strain KCOM 1350 (= ChDC B183), complete genome. Accession number CP012646.1. https://www.ncbi.nlm.nih.gov/nuccore/CP012646.1 GenBank: *Streptococcus mitis* strain SVGS_061 chromosome, complete genome. Accession number CP014326.1. https://www.ncbi.nlm.nih.gov/nuccore/CP014326.1 GenBank: *Streptococcus mitis* NCTC 12261 chromosome, complete genome. Accession number CP028414.1. https://www.ncbi.nlm.nih.gov/nuccore/CP028414.1 GenBank: *Streptococcus oralis* strain NCTC11427, chromosome 1, complete genome. Accession number LR134336.1. https://www.ncbi.nlm.nih.gov/nuccore/LR134336.1 GenBank: *Streptococcus oralis* strain 34 chromosome, complete genome. Accession number CP079724.1. https://www.ncbi.nlm.nih.gov/nuccore/CP079724.1 GenBank: *Streptococcus oralis* strain FDAARGOS_886 chromosome, complete genome. Accession number CP065706.1. https://www.ncbi.nlm.nih.gov/nuccore/CP065706.1 GenBank: *Streptococcus oralis* subsp. *dentisani* strain F0392 chromosome, complete genome. Accession number CP034442.1. https://www.ncbi.nlm.nih.gov/nuccore/CP034442.1 GenBank: *Streptococcus pneumoniae* strain 475 chromosome, complete genome. Accession number CP046355.1. https://www.ncbi.nlm.nih.gov/nuccore/CP046355.1 GenBank: *Streptococcus pneumoniae* NU83127 DNA, complete genome. Accession number AP018936.1. https://www.ncbi.nlm.nih.gov/nuccore/AP018936.1 GenBank: *Streptococcus pneumoniae* NCTC7465, chromosome 1, complete genome. Accession number LN831051.1. https://www.ncbi.nlm.nih.gov/nuccore/LN831051.1 GenBank: *Streptococcus pneumoniae* strain 6A-10 chromosome, complete genome. Accession number CP053210.1. https://www.ncbi.nlm.nih.gov/nuccore/CP053210.1 GenBank: *Streptococcus mitis* strain 127R, partial 16S rRNA gene. Accession number AJ295848.1. https://www.ncbi.nlm.nih.gov/nuccore/AJ295848.1 GenBank: *Streptococcus mitis* clone 2C4, 16S rRNA gene. Accession number AM157428.1. https://www.ncbi.nlm.nih.gov/nuccore/AM157428.1 GenBank: *Streptococcus mitis* strain NS51, partial 16S rRNA gene. Accession number NR_028664.1. https://www.ncbi.nlm.nih.gov/nuccore/NR_028664.1 GenBank: *Streptococcus mitis* bv. 2 strain F0392, partial 16S rRNA gene. Accession number GU470907.1. https://www.ncbi.nlm.nih.gov/nuccore/GU470907.1 GenBank: *Streptococcus mitis* strain ChDC B553, partial 16S rRNA gene. Accession number KF933785. https://www.ncbi.nlm.nih.gov/nuccore/KF933785.1 GenBank: *Streptococcus mitis* strain FC6528, partial 16S rRNA gene. Accession number OM368574.1. https://www.ncbi.nlm.nih.gov/nuccore/OM368574.1 GenBank: *Streptococcus pneumoniae* strain FC6532, partial 16S rRNA gene. Accession number OM368578.1. https://www.ncbi.nlm.nih.gov/nuccore/OM368578.1 GenBank: *Streptococcus pneumoniae* clone 4V4, 16S rRNA gene. Accession number AM157442. https://www.ncbi.nlm.nih.gov/nuccore/AM157442.1 GenBank: *Streptococcus oralis* subsp. *dentisani* strain 7747, partial 16S rRNA gene. Accession number NR_117719. https://www.ncbi.nlm.nih.gov/nuccore/NR_117719.1 GenBank: *Enterobacter asburiae* strain ATCC 35953 chromosome, complete genome. Accession number NZ_CP011863. https://www.ncbi.nlm.nih.gov/nuccore/NZ_CP011863.1 GenBank: *Streptococcus mitis* strain HAC11, isolate #11, partial 16S rRNA gene. Accession number LT707617. https://www.ncbi.nlm.nih.gov/nuccore/LT707617.1 GenBank: *Streptococcus mitis* strain NCTC 3165, MAFF 911479, 16S rRNA gene. Accession number AB002520.1. https://www.ncbi.nlm.nih.gov/nuccore/AB002520.1

## References

[ref1] Alachiotis N Vogiatzi E Pavlidis P : Chromatogate: a tool for detecting base mis-calls in multiple sequence alignments by semi-automatic chromatogram inspection. *Comput. Struct. Biotechnol. J.* 2013;6:e201303001. 10.5936/csbj.201303001 24688709 PMC3962156

[ref2] Altschul SF Gish W Miller W : Basic local alignment search tool. *J. Mol. Biol.* 1990;215:403–410. 10.1016/S0022-2836(05)80360-2 2231712

[ref3] Bagheri H Severin AJ Rajan H : Detecting and correcting misclassified sequences in the large-scale public databases. *Bioinformatics.* 2020;36:4699–4705. 10.1093/bioinformatics/btaa586 32579213 PMC7821992

[ref4] Baltrus DA : Divorcing strain classification from species names. *Trends Microbiol.* 2016;24:431–439. 10.1016/j.tim.2016.02.004 26947794

[ref5] Benitez-Paez A Sanz Y : Multi-locus and long amplicon sequencing approach to study microbial diversity at species level using the MinIONTM portable Nanopore sequencer. *Gigascience.* 2017;6:1–12. 10.1093/gigascience/gix043 28605506 PMC5534310

[ref6] Boudewijns M Bakkers JM Sturm PDJ : 16S rRNA gene sequencing and the routine clinical microbiology laboratory: A perfect marriage? *J. Clin. Microbiol.* 2006;44:3469–3470. 10.1128/JCM.01017-06 16954306 PMC1594676

[ref7] Church DL Cerutti L Gürtler A : Performance and application of 16S rRNA gene cycle sequencing for routine identification of bacteria in the clinical microbiology laboratory. *Clin. Microbiol. Rev.* 2020;33:e00053–e00019. 10.1128/CMR.00053-19 32907806 PMC7484979

[ref8] Ciccarelli FD Doerks T Mering C von : Toward automatic reconstruction of a highly resolved tree of life. *Science.* 2006;311:1283–1287. 10.1126/science.1123061 16513982

[ref9] Clarridge JE : Impact of 16S rRNA gene sequence analysis for identification of bacteria on clinical microbiology and infectious diseases. *Clin. Microbiol. Rev.* 2004;17:840–862. 10.1128/CMR.17.4.840-862.2004 15489351 PMC523561

[ref10] Deurenberg RH Bathoorn E Chlebowicz MA : Application of next generation sequencing in clinical microbiology and infection prevention. *J. Biotechnol.* 2017;243:16–24. 10.1016/j.jbiotec.2016.12.022 28042011

[ref11] Devanga-Ragupathi NK Muthuirulandi SDP Inbanathan FY : Accurate differentiation of *Escherichia coli* and *Shigella* serogroups: challenges and strategies. *New Microbes New Infect.* 2018;21:58–62. 10.1016/j.nmni.2017.09.003 29204286 PMC5711669

[ref12] Devulder G Montclos MP de Flandrois JP : A multigene approach to phylogenetic analysis using the genus *Mycobacterium* as a model. *Int. J. Syst. Evol. Microbiol.* 2005;55:293–302. 10.1099/ijs.0.63222-0 15653890

[ref13] Ibal JC Pham HQ Park CE : Information about variations in multiple copies of bacterial 16S rRNA genes may aid in species identification. *PLoS One.* 2019;14:e0212090. 10.1371/journal.pone.0212090 30768621 PMC6377111

[ref14] Janda JM Abbott SL : 16S rRNA gene sequencing for bacterial identification in the diagnostic laboratory: Pluses, perils, and pitfalls. *J. Clin. Microbiol.* 2007;45:2761–2764. 10.1128/JCM.01228-07 17626177 PMC2045242

[ref15] Johnson JS Spakowicz DJ Hong BY : Evaluation of 16S rRNA gene sequencing for species and strain-level microbiome analysis. *Nat. Commun.* 2019;10:5011–5029. 10.1038/s41467-019-13036-1 31695033 PMC6834636

[ref16] Johnston PR Quijada L Smith CA : A multigene phylogeny toward a new phylogenetic classification of *Leotiomycetes.* *IMA Fungus.* 2019;10:1. 10.1186/s43008-019-0002-x 32647610 PMC7325659

[ref17] Kerkhof LJ Dillon KP Haggblom MM : Profiling bacterial communities by MinION sequencing of ribosomal operons. *Microbiome.* 2017;5:116. 10.1186/s40168-017-0336-9 28911333 PMC5599880

[ref18] Kumar S Stecher G Li M : MEGA X: Molecular evolutionary genetics analysis across computing platforms. *Mol. Biol. Evol.* 2018;35:1547–1549. 10.1093/molbev/msy096 29722887 PMC5967553

[ref19] Lal D Verma M Lal R : Exploring internal features of 16S rRNA gene for identification of clinically relevant species of the genus *Streptococcus.* *Ann. Clin. Microbiol. Antimicrob.* 2011;10:28. 10.1186/1476-0711-10-28 21702978 PMC3151204

[ref20] Leray M Knowlton N Ho SL : GenBank is a reliable resource for 21 ^st^ century biodiversity research. *Proc. Natl. Acad. Sci. USA.* 2019;116:22651–22656. 10.1073/pnas.1911714116 31636175 PMC6842603

[ref21] Liu Y Lai Q Shao Z : Genome analysis-based reclassification of *Bacillus weihenstephanensis* as a later heterotypic synonym of *Bacillus mycoides.* *Int. J. Syst. Evol. Microbiol.* 2018;68:106–112. 10.1099/ijsem.0.002466 29095136

[ref22] Martínez-Romero E Rodríguez-Medina N Beltrán-Rojel M : Genome misclassification of *Klebsiella variicola* and *Klebsiella quasipneumoniae* isolated from plants, animals and humans. *Salud Publica Mex.* 2018;60:56–62. 10.21149/8149 29689657

[ref23] Mateo-Estrada V Grana-Miraglia L Lopez-Leal G : Phylogenomics reveals clear cases of misclassification and genus-wide phylogenetic markers for *Acinetobacter.* *Genome Biol. Evol.* 2019;11:2531–2541. 10.1093/gbe/evz178 31406982 PMC6740150

[ref24] Parks DH Waite DW Skarshewski A : A standardized bacterial taxonomy based on genome phylogeny substantially revises the tree of life. *Nat. Biotechnol.* 2018;36:996–1004. 10.1038/nbt.4229 30148503

[ref25] Paul B Dixi G Murali TS : Genome-based taxonomic classification. *Genome.* 2019;62:45–52. 10.1139/gen-2018-0072 30649978

[ref26] Paul B : Concatenated 16S rRNA sequence analysis improves bacterial taxonomy. 2022. 10.51281/zenodo.7384709 PMC10521043 37767069

[ref27] Peker N Garcia-Croes S Dijkhuizen B : A comparison of three different bioinformatics analyses of the 16S-23S rRNA encoding region for bacterial identification. *Front. Microbiol.* 2019;10:620. 10.3389/fmicb.2019.00620 31040829 PMC6476902

[ref28] Quast C Pruesse E Yilmaz P : The SILVA ribosomal RNA gene database project: improved data processing and web-based tools. *Nucleic Acids Res.* 2013;41:D590–D596. 10.1093/nar/gks1219 23193283 PMC3531112

[ref29] Reller LB Weinstein MP Petti CA : Detection and identification of microorganisms by gene amplification and sequencing. *Clin. Infect. Dis.* 2007;44:1108–1114. 10.1086/512818 17366460

[ref30] Sabat AJ Zanten E van Akkerboom V : Targeted next-generation sequencing of the 16S-23S rRNA region for culture-independent bacterial identification increased discrimination of closely related species. *Sci. Rep.* 2017;7:1–12. 10.1038/s41598-017-03458-6 28611406 PMC5469791

[ref31] Schloss PD : Reintroducing mothur: 10 Years Later. *Appl. Environ. Microbiol.* 2020;86:e02343–e02319. 10.1128/AEM.02343-19 31704678 PMC6952234

[ref32] Sievers F Wilm A Dineen D : Fast, scalable generation of high-quality protein multiple sequence alignments using Clustal Omega. *Mol. Syst. Biol.* 2011;7:539. 10.1038/msb.2011.75 21988835 PMC3261699

[ref33] Srinivasan R Karaoz U Volegova M : Use of 16S rRNA gene for identification of a broad range of clinically relevant bacterial pathogens. *PLoS One.* 2015;10:e0117617. 10.1371/journal.pone.0117617 25658760 PMC4319838

[ref34] Stackebrandt E Mondotte JA Fazio LL : Authors need to be prudent when assigning names to microbial isolates. *Arch. Microbiol.* 2021;203:5845–5848. 10.1007/s00203-021-02599-7 34709418

[ref35] Starke R Pylro VS Morais DK : 16S rRNA gene copy number normalization does not provide more reliable conclusions in metataxonomic surveys. *Microb. Ecol.* 2021;81:535–539. 10.1007/s00248-020-01586-7 32862246 PMC7835310

[ref36] Steven B Hesse C Soghigian J : Simulated rRNA/DNA ratios show potential to misclassify active populations as dormant. *Appl. Environ. Microbiol.* 2017;83:e00696–e00617. 10.1128/AEM.00696-17 28363969 PMC5440720

[ref37] Thiergart T Landan G Martin WF : Concatenated alignments and the case of the disappearing tree. *BMC Evol. Biol.* 2014;14:212–266. 10.1186/s12862-014-0266-0 25547755 PMC4302582

[ref38] Varghese NJ Mukherjee S Ivanova N : Microbial species delineation using whole genome sequences. *Nucleic Acids Res.* 2015;43:6761–6771. 10.1093/nar/gkv657 26150420 PMC4538840

[ref39] Vetrovsky T Baldrian P : The variability of the 16S rRNA gene in bacterial genomes and its consequences for bacterial community analyses. *PLoS One.* 2013;8:e57923. 10.1371/journal.pone.0057923 23460914 PMC3583900

[ref40] Weisburg WG Barns SM Pelletier DA : 16S ribosomal DNA amplification for phylogenetic study. *J. Bacteriol.* 1991;173:697–703. 10.1128/jb.173.2.697-703.1991 1987160 PMC207061

[ref41] Winand R Bogaerts B Hoffman S : Targeting the 16S rRNA gene for bacterial identification in complex mixed samples: Comparative evaluation of second (Illumina) and third (Oxford Nanopore technologies) generation sequencing technologies. *Int. J. Mol. Sci.* 2020;21:298. 10.3390/ijms21010298 31906254 PMC6982111

[ref42] Yoon SH Ha SM Kwon S : Introducing EzBioCloud: a taxonomically united database of 16S rRNA gene sequences and whole-genome assemblies. *Int. J. Syst. Evol. Microbiol.* 2017;67:1613–1617. 10.1099/ijsem.0.001755 28005526 PMC5563544

