# Concatenated 16S rRNA sequence analysis improves bacterial taxonomy

**DOI:** 10.12688/f1000research.128320.3

**Published:** 2023-09-01

**Authors:** Bobby Paul

**Affiliations:** 1 Department of Bioinformatics, Manipal School of Life Sciences, Manipal Academy of Higher Education, Manipal, Karnataka, 576104, India

**Keywords:** bacterial nomenclature, bacterial taxonomy, concatenated phylogeny, species-specific barcode reference library

## Abstract

**Background: **Microscopic, biochemical, molecular, and computer-based approaches are extensively used to identify and classify bacterial populations. Advances in DNA sequencing and bioinformatics workflows have facilitated sophisticated genome-based methods for microbial taxonomy although sequencing of the 16S rRNA gene is widely employed to identify and classify bacterial communities as a cost-effective and single-gene approach. However, the 16S rRNA sequence-based species identification accuracy is limited because of the occurrence of multiple copies of the 16S rRNA gene and higher sequence identity between closely related species. The availability of the genomes of several bacterial species provided an opportunity to develop comprehensive species-specific 16S rRNA reference libraries.

**Methods:** Sequences of the 16S rRNA genes were retrieved from the whole genomes available in the Genome databases. With defined criteria, four 16S rRNA gene copy variants were concatenated to develop a species-specific reference library. The sequence similarity search was performed with a web-based BLAST program, and MEGA software was used to construct the phylogenetic tree.

**Results:** Using this approach, species-specific 16S rRNA gene libraries were developed for four closely related *Streptococcus* species ( *S. gordonii*, *S. mitis*, *S. oralis*, and *S. pneumoniae*). Sequence similarity and phylogenetic analysis using concatenated 16S rRNA copies yielded better resolution than single gene copy approaches.

**Conclusions:** The approach is very effective in classifying genetically closely related bacterial species and may reduce misclassification of bacterial species and genome assemblies.

## Introduction

The genomic region encoding the 16S ribosomal RNA (16S rRNA) is extensively studied, and used to identify and classify bacterial species. The 16S rRNA is a conserved component of the small subunit (30S) of the prokaryotic ribosome. The gene encoding the 16S rRNA is ~1500 base pair (bp) long, and it consists of nine variable regions ( Reller *et al*. 2007; Chakravorty *et al.* 2007; Sabat *et al*. 2017). The sequence of the 16S rRNA gene has been extensively used as a molecular marker in culture-independent methods to identify and classify diverse bacterial communities ( Clarridge 2004; Johnson *et al*. 2019). Bacterial 16S rRNA sequences are currently being used to study the evolution, phylogenetic relationships, and environmental abundance of various taxa ( Vetrovsky and Baldrian 2013; Srinivasan *et al*. 2015; Peker *et al*. 2019).

Although 16S rRNA sequence analyses are the mainstay of taxonomic studies of bacteria, there are some limitations. For example, the 16S rRNA gene has poor discriminatory power at the species level ( Winand *et al*. 2020), and the copy number per genome can vary from 1 to 15 or even more ( Vetrovsky and Baldrian 2013; Winand *et al*. 2020). The variable copies of this gene within a genome makes distinct data for a species. Therefore, gene copy normalization (GCN) may be necessary prior to sequence analysis. However, GCN may not improve the 16S rRNA sequence analyses in all scenarios, and comprehensive, species-specific catalogues of 16S rRNA gene copies may be necessary ( Starke *et al*. 2021). Furthermore, intra-species variations in the 16S rRNA gene copies were observed in several bacterial genome assemblies ( Paul *et al*. 2019). Only a few bacterial species contain identical 16S rRNA gene copies, and sequence diversity increases with increasing copy numbers of 16S rRNA genes ( Vetrovsky and Baldrian 2013). The high levels of similarity of the 16S rRNA gene across some bacterial species poses a major challenge for taxonomic studies using bioinformatics methods ( Deurenberg *et al*. 2017; Peker *et al*. 2019).

Factors such as purity of bacterial cultures, quality of the purified DNA samples, and potential DNA chimeras should be carefully considered while sequencing and analysis of 16S rRNA genes ( Janda and Abbott 2007; Church *et al*. 2020). Sequencing errors can lead to misidentification of bacteria and phylogenetic anomalies ( Alachiotis *et al*. 2013). Other concerns include sequence ambiguities, gaps generated during DNA sequencing and sequence comparisons, and choosing the appropriate algorithm (local or global) for sequence alignment. Since the local alignment algorithm is extensively used for sequence similarity-based comparisons, it is important to carefully consider whether a single variable region or a combination of variable regions of the 16S rRNA gene would be ideal for bacterial classification ( Janda and Abbott 2007; Johnson *et al*. 2019; Winand *et al*. 2020). Using erroneous 16S rRNA sequences as references and improper bioinformatics workflows can mislead bacterial identification. Further, the growth of bioinformatics and genetic data has led to the current genome-based microbial classification. However, the success rate of these approaches are highly dependent on the skill of data analyst personnel in next generation sequencing technologies, computational tools, operation of high performance computing systems. Researchers without sufficient experience or skill in such technologies may also mislead the bacterial taxonomy ( Baltrus 2016).

Other methods for bacterial identification include the sequencing and analysis of the polymerase chain reaction (PCR) amplified ∼4.5 kb 16S–23S rRNA regions ( Benitez-Paez and Sanz 2017; Sabat *et al*. 2017; Kerkhof *et al*. 2017). However, the 16S–23S rRNA sequence-based method is less practical application due to the lack of appropriate reference sequence databases and reliable tools/methods for sequence analysis ( Sabat *et al*. 2017). Recent advances in bioinformatics workflows ( Winand *et al*. 2020; Schloss 2020) and reference databases such as SILVA, EzBioCloud ( Quast *et al*. 2013; Yoon *et al*. 2017) have further improved 16S rRNA-based bacterial taxonomy. However, these approaches are not completely reliable due to misclassification of some bacterial species and erroneous genome assemblies ( Steven *et al*. 2017; Martínez-Romero *et al*. 2018; Mateo-Estrada *et al*. 2019; Bagheri *et al*. 2020).

The entire 16S rRNA gene (~1500 bp) can be amplified and sequenced using the conventional or high throughput sequencing methods. However, many 16S rRNA sequence-based bacterial identification studies do not seem to include all of these nine variable regions ( Stackebrandt *et al*. 2021). Due to the large volume of whole-genome data that is being produced by high throughput sequencing technologies, there is an urgent need to translate the genomic data for convenient microbiome analyses that ensure clinical practitioners can readily understand and quickly implement ( Church *et al*. 2020). This study aimed to develop a workflow for accurate identification of bacteria using concatenated, species-specific 16S rRNA sequences. It was hoped that the species-specific libraries would yield much better resolution in sequence similarity- and phylogeny-based bacterial classification.

## Methods

### Estimation of variations in intra-genomic 16S rRNA gene copies

It has been reported that sequence alignment of 16S rRNA gene copies at the intra-genomic level shows a higher degree of variability in species belonging to the *Firmicutes* and *Proteobacteria* ( Vetrovsky and Baldrian 2013; Ibal *et al*. 2019). Therefore, this study used eight 16S rRNA gene copies (Underlying data: Supplementary data 1 ( Paul 2022)) retrieved from the complete genome of *Enterobacter asburiae* strain ATCC 35953 (NZ_CP011863.1). To estimate intra-genomic variability between these 16S rRNA gene copies, BLAST+ 2.13.0 (RRID:SCR_004870; Altschul *et al*. 1990) and Clustal Omega 1.2.4 (RRID:SCR_001591; Sievers *et al*. 2011) sequence alignment algorithms were used. Previous studies suggested unweighted pair group method with arithmetic averages (UPGMA) algorithm for the phylogenetic analysis of 16S rRNA genes ( Clarridge 2004; Caporaso *et al*. 2011). Hence, phylogenetic analysis of these 16S rRNA gene copies were performed using the UPGMA method (Maximum Composite Likelihood; 500 bootstrap replicates) provided in the MEGA software (version 11; RRID: SCR_000667; Kumar *et al*. 2018).

### Construction of species-specific concatenated 16S rRNA reference libraries

Previous studies have reported that the genes encoding 16S rRNA from several bacterial species share >99% sequence identity ( Deurenberg *et al*. 2017; Peker *et al*. 2019). Therefore, the 16S rRNA-based methods failed to correctly identify bacterial species that are genetically closely related ( Deurenberg *et al*. 2017; Devanga-Ragupathi *et al*. 2018). It has been reported that 16S rRNA-based methods cannot distinguish between *Streptococcus mitis* and *Streptococcus pneumoniae* due to the high sequence similarity ( Reller *et al*. 2007; Lal *et al*. 2011). Hence, the study decided to choose the 16S rRNA gene copies from four closely related species of *Streptococcus.*

More than 552,575 whole-genome sequences are currently (Aug 2023) available for bacterial species in the Genome database (RRID:SCR_002474; https://www.ncbi.nlm.nih.gov/genome). Many of these genomes were sequenced using high throughput sequencing technologies such as Illumina/Ion-Torrent (short read sequencing) and PacBio/Nanopre (long read sequencing). Furthermore, most of these whole-genome sequences were obtained after a hybrid assembly of short and long read sequence data. This extensive, high throughput data can be effectively used to develop advanced genome-based methods for microbial systematics. Although the genomic data is available in four levels (contig, scaffold, chromosome, and complete), this study used only the complete genomes to retrieve 16S rRNA genes.

To develop species-specific barcode reference libraries, this study retrieved full-length 16S rRNA genes from 16 complete genome sequences belonging to four *Streptococcus* species ( *S. gordonii*, *S. mitis*, *S. oralis,* and *S. pneumoniae*). Details of the dataset used to develop species-specific concatenated reference libraries are provided in Table 1, and the sequences are provided in the underlying data (Supplementary data 2 ( Paul 2022)). Sequences were trimmed beyond the universal primer pair (fD1-5′-GAG TTT GAT CCT GGC TCA-3′ and rP2-5′-ACG GCT AAC TTG TTA CGA CT-3′, which are used for full-length 16S rDNA amplification, Weisburg *et al*. 1991) to maintain uniform length. To perform multiple sequence alignment and identify the intra-species parsimony informative (Parsim-info) variable sites, the MEGA 11 software was used. A species-specific barcode reference library that covers the entire Parsim-info variable sites was constructed by concatenating four 16S rRNA gene copies from four different strains of a species. The rationale for the selection of four copies for constructing a species-specific barcode reference library was: (i) a maximum of four variations can be found at a single site, and (ii) earlier studies have shown that the mean 16S rRNA copies per genome is four ( Vetrovsky and Baldrian 2013).

**Table 1.** Details of whole genome assemblies used for the development of concatenated 16S rRNA reference libraries. One copy of 16S rRNA gene from each strain is used for the concatenation.

Species	Strains	Genome accession number	No. of 16S rRNA gene copies	Sequencing platform	Species-specific library name	Library length (bp)	No. of Parsim-info sites
*S. gordonii*	FDAARGOS 1454	CP077224.1	4	PacBio; Illumina	*S.gordonii-*Ref-I	6076	7
	NCTC7868	LR134291.1	4	PacBio			
	KCOM 1506	CP012648.1	5	Illumina			
	NCTC9124	LR594041.1	4	PacBio			
*S. mitis*	B6	NC_013853.1	4	NA	*S.mitis-*Ref-I	6033	10
	KCOM 1350	CP012646.1	3	Illumina			
	SVGS 061	CP014326.1	4	PacBio; Illumina			
	NCTC 12261	CP028414.1	4	PacBio			
*S. oralis*	NCTC 11427	LR134336.1	4	PacBio	*S.oralis-*Ref-I	6038	24
	34	CP079724.1	4	Illumina; Nanopore			
	FDAARGOS 886	CP065706.1	4	PacBio; Illumina			
	F0392	CP034442.1	4	PacBio			
*S. pneumoniae*	475	CP046355.1	4	PacBio	*S.pneumoniae*-Ref-I	6032	6
	NU83127	AP018936.1	4	Nanopore; Illumina			
	NCTC7465	LN831051.1	4	PacBio			
	6A-10	CP053210.1	4	PacBio			

### Demonstration of concatenated 16S rRNA in sequence similarity and phylogeny

This study analyzed a few cases to demonstrate (i) the classical sequence similarity and (ii) phylogenetic analysis using concatenated species-specific 16S rRNA reference libraries. The study used nine 16S rRNA gene copies (sequenced using the Sanger method) showing higher sequence similarity to the 16S rRNA genes of multiple species of *Streptococcus* were retrieved from GenBank database (RRID:SCR_002760). The web-based BLAST2 (version 2.13.0) program for aligning two or more sequences was used to estimate the maximum score, total alignment score, and sequence identity of these nine 16S rRNA sequences selected. For the sequence similarity search, a single copy of the 16S rRNA (sequenced using the Sanger method or retrieved from a whole-genome assembly) can be considered as ‘Query sequence’. The concatenated species-specific reference libraries need to be provided in the text area for ‘Subject sequence’. However, to perform phylogenetic analysis, it is mandatory that the target sequence (length = n bp) be concatenated four times (length = 4 × n bp). Phylogenetic analysis was performed for single gene copies and concatenated approach using UPGMA method as indicated above.

## Results

### Intra-genomic 16S rRNA variations in *E. asburiae*

**Figure 1.**
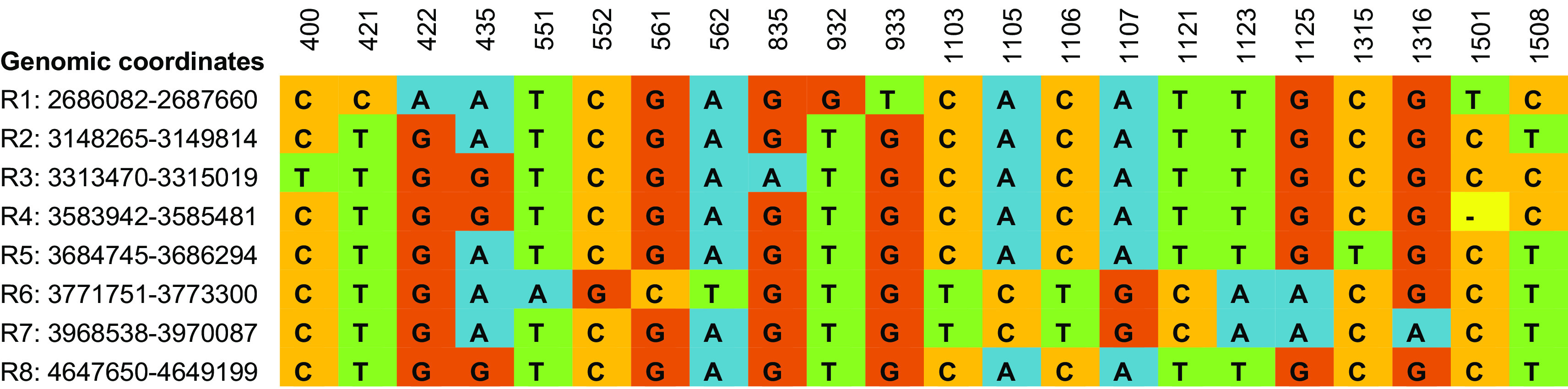
Clustal Omega based multiple sequence alignment of eight intra genomic 16S rRNA gene copies from *Enterobacter asburiae* strain ATCC 35953 (NZ_CP011863.1) showing 22 variable sites. According to Chakravorty *et al.* (2007), the nine variable regions of 16S rRNA gene spanned nucleotides 69-99, 137-242, 433-497, 576-682, 822-879, 986-1043, 1117-1173, 1243-1294, and 1435-1465 for V1 to V9 respectively.

Historically, sequences of the 16S rRNA genes have been used to identify known and new bacterial species. However, efficiency of PCR-based amplification, poor discrimination at the species level, multiple polymorphic 16S rRNA gene copies, and improper bioinformatics workflows for the data analysis can impact the identification. The genome of *E. asburiae* contains eight copies of the 16S rRNA gene. Analysis using Clustal Omega (global alignment) and BLAST (local alignment) showed that the sequences of these eight alleles had average identities of 99.29 and 99%, respectively ( Table 2). Therefore, choosing the appropriate algorithm/tool is critical for the estimation of sequence identities and sequence-based species delineation. For analyzing sequence pairs that are highly identical, global sequence alignment algorithms seem to be more appropriate because they consider all the nucleotides for the estimation of sequence identity. Clustal Omega based multiple sequence alignment of the eight alleles of the 16S rRNA gene in the genome of *E. asburiae* showed 22 variable sites ( Figure 1). These results show that the computational analysis using a single gene copy makes different results for species harbouring variable copies of this gene.

**Table 2.** Percent identity of eight intra genomic 16S rRNA regions from *Enterobacter asburiae* strain ATCC 35953 (NZ_CP011863.1). Percent identity given below the diagonal line is calculated with Clustal Omega software (Mean identity: 99.29%) and those above the diagonal line were calculated with the BLASTN program (Mean identity: 99.00%). Genome coordinates of 16S rRNA copies: R1: 2686082–2687660 (1579 bp); R2: 3148265–3149814 (1550 bp); R3: 3313470–3315019 (1550 bp); R4: 3583942–3585481 (1540 bp); R5:3684745–3686294 (1550 bp); R6: 3771751–3773300 (1550 bp); R7: 3968538–3970087 (1550 bp); R8: 4647650–4649199 (1550 bp).

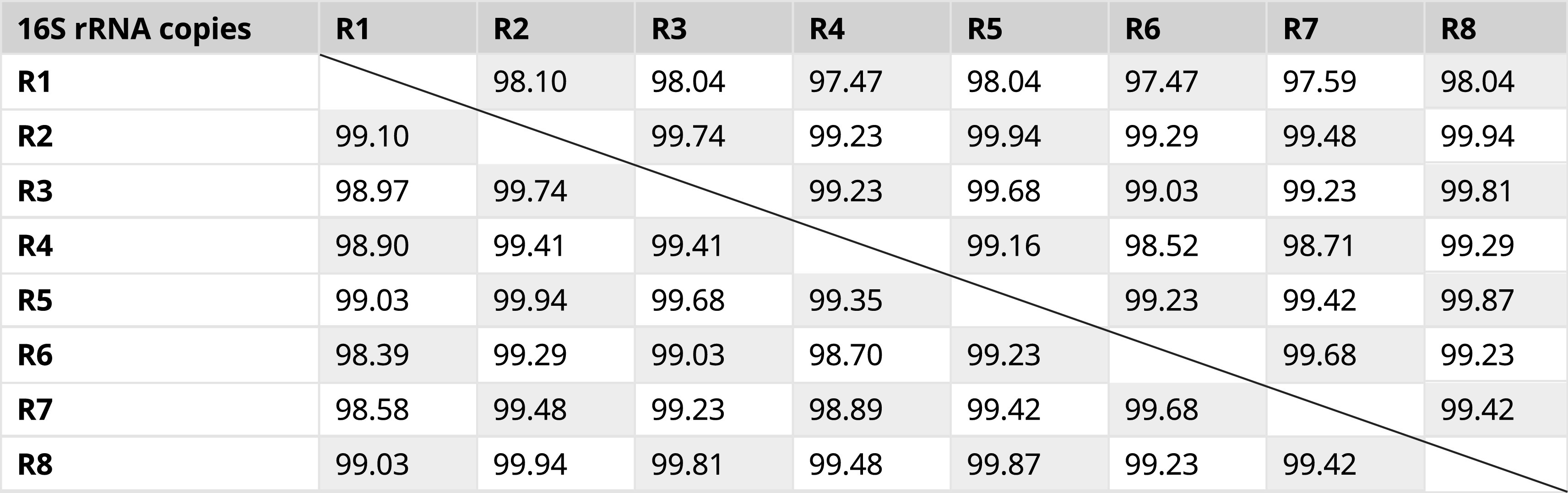

**Figure 2.**
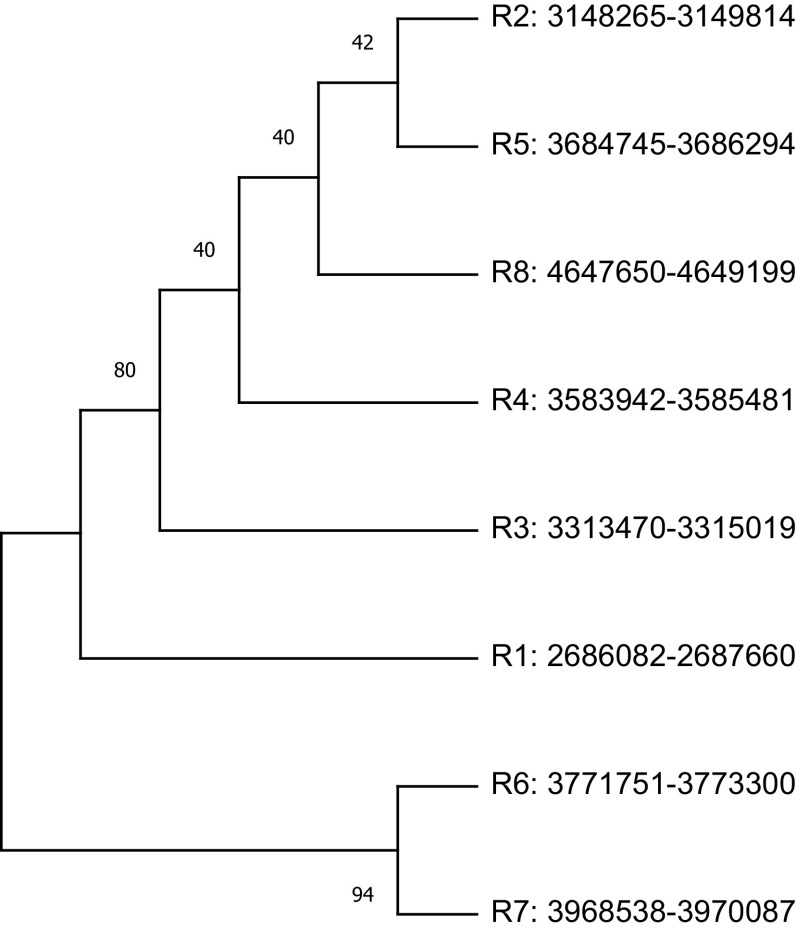
Phylogenetic tree of eight intra genomic 16S rRNA gene copies from *Enterobacter asburiae* strain ATCC 35953 (NZ_CP011863.1). The node label denotes the coordinate of 16S rRNA regions in the genome.

The evolutionary relationship between species is usually represented using a phylogenetic tree based on the analysis of a single gene, multiple genes, or whole genomes. However, bacterial identification and classification is mainly based on the phylogenetic analysis of single copies of 16S rRNA genes. A phylogenetic tree was constructed to understand how variations in the sequences of the eight alleles of the 16S rRNA gene in the genome of *E. asburiae* influence species delineation ( Figure 2). These results indicate that the intra-genomic variations in 16S rRNA copies may mislead the bacterial taxonomy in single gene copy approaches.

### Species-specific concatenated 16S rRNA libraries

This study selected four species of *Streptococcus* ( *S. gordonii*, *S. mitis*, *S. oralis*, and *S. pneumoniae*) to construct species-specific concatenated reference libraries based on 16S rRNA gene sequences obtained from complete genomes. Four variable copies of the 16S rRNA gene from a species are required to construct a species-specific concatenated reference library. The details of species-specific libraries are listed in Table 1 and the sequences are provided in the underlying data (Supplementary data 3 ( Paul 2022)). Analysis using the sequences of 16S rRNA genes showed 24, 10, 7, and 6 Parsim-info variable sites for *S. oralis*, *S. mitis*, *S. gordonii,* and *S. pneumoniae*, respectively *.* The intra-species Parsim-info variable sites were located in both the conserved and variable regions of the 16S rRNA gene (Supplementary data 4 ( Paul 2022)).

The study used full-length 16S rRNA gene copies from four different strains to highlight the variations at the species level. However, a large number of partial 16S rRNA gene sequences are available in the public genetic databases. Further, many researchers are amplifying only few variable regions of the 16S rRNA gene. In such cases, a species-specific concatenated reference library can be constructed using partial sequences. Intra-species variations in the sequences of 16S rRNA gene copies influence the sequence-based bacterial identification. Therefore, concatenation of the sequences of 16S rRNA gene provides much better resolution compared to analysis using sequences from a single copy of the 16S rRNA gene.

### Demonstration of concatenated 16S rRNA based species identification

This study compared sequences of nine 16S rRNA genes from different species of *Streptococcus* ( Table 3) against the species-specific concatenated reference libraries constructed. The analysis showed that the concatenated sequences provide much better resolution in sequence similarity search and phylogenetic analysis. The sequence accession numbers GU470907.1 and KF933785.1 classified as *S. mitis* showed a higher maximum and total alignment score with concatenated 16S rRNA library of *S. oralis* than *S. mitis* ( Table 3). Two sequences (OM368574.1 classified as *S. mitis* and OM368578.1 classified as *S. pneumoniae*) showed same score against the four reference libraries constructed. Based on the maximum total alignment score these two sequences are belonging to *S. pneumoniae*, however, they classified as two separate species. Interestingly, the sequence GU470907.1 classified as *S. mitis* showed 100% identity with *S. oralis* reference library with a total alignment score of 10936.

**Table 3.** Similarity of selected sequences against the concatenated species-specific 16S rRNA reference libraries.

GenBank Accession Number	Species	*S. gordonii*-Ref-I			*S. mitis*-Ref-I			*S. oralis*-Ref-I			*S. pneumoniae*-Ref-I		
		Max Score	Total Score	Identity (%)	Max Score	Total Score	Identity (%)	Max Score	Total Score	Identity (%)	Max Score	Total Score	Identity (%)
AJ295848.1	*S. mitis*	2495	9967	96.45	2769	11027	99.80	2758	10851	99.67	2752	10982	99.60
AM157428.1	*S. mitis*	2462	9845	96.05	2724	10866	99.27	2702	10685	99.01	2708	10805	99.07
NR_028664.1	*S. mitis*	2499	9991	96.45	2776	10979	99.87	2750	10864	99.54	2724	10888	99.27
GU470907.1	*S. mitis*	2536	10096	96.91	2715	10796	99.14	2787	10936	100	2091	10716	98.87
KF933785.1	*S. mitis*	2466	9832	96.06	2667	10593	98.54	2673	10650	98.61	2632	10502	98.15
OM368574.1	*S. mitis*	2475	9896	96.24	2754	10968	99.67	2732	10814	99.40	2760	10990	99.73
OM368578.1	*S. pneumoniae*	2475	9896	96.24	2754	10968	99.67	2732	10814	99.40	2760	10990	99.73
AM157442.1	*S. pneumoniae*	2470	9863	96.12	2702	10779	99.01	2715	10726	99.14	2702	10777	99.01
NR_117719.1	*S. oralis*	2531	10074	96.84	2710	10774	99.07	2787	10925	100	2697	10739	98.94

**Figure 3.**
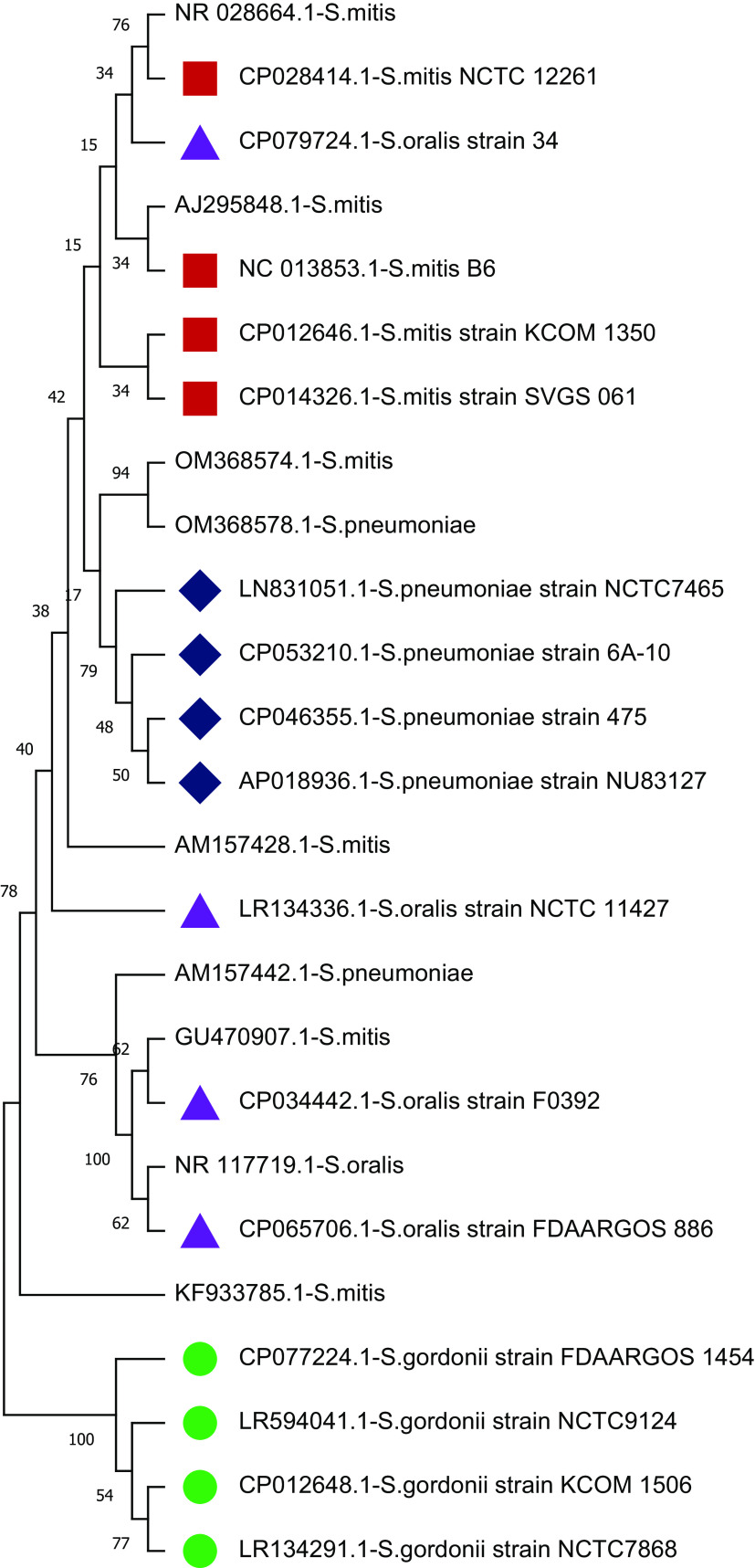
Phylogenetic analysis of randomly selected nine 16S rRNA sequences classified as *Streptococcus* species and sequences used for species-specific reference library. The phylogenetic tree plotted using single copy approach. The node name highlighted in shapes ( 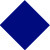, 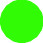, 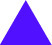, 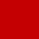) represents the sequences which are used for the construction of four concatenated species-specific reference libraries.

**Figure 4.**
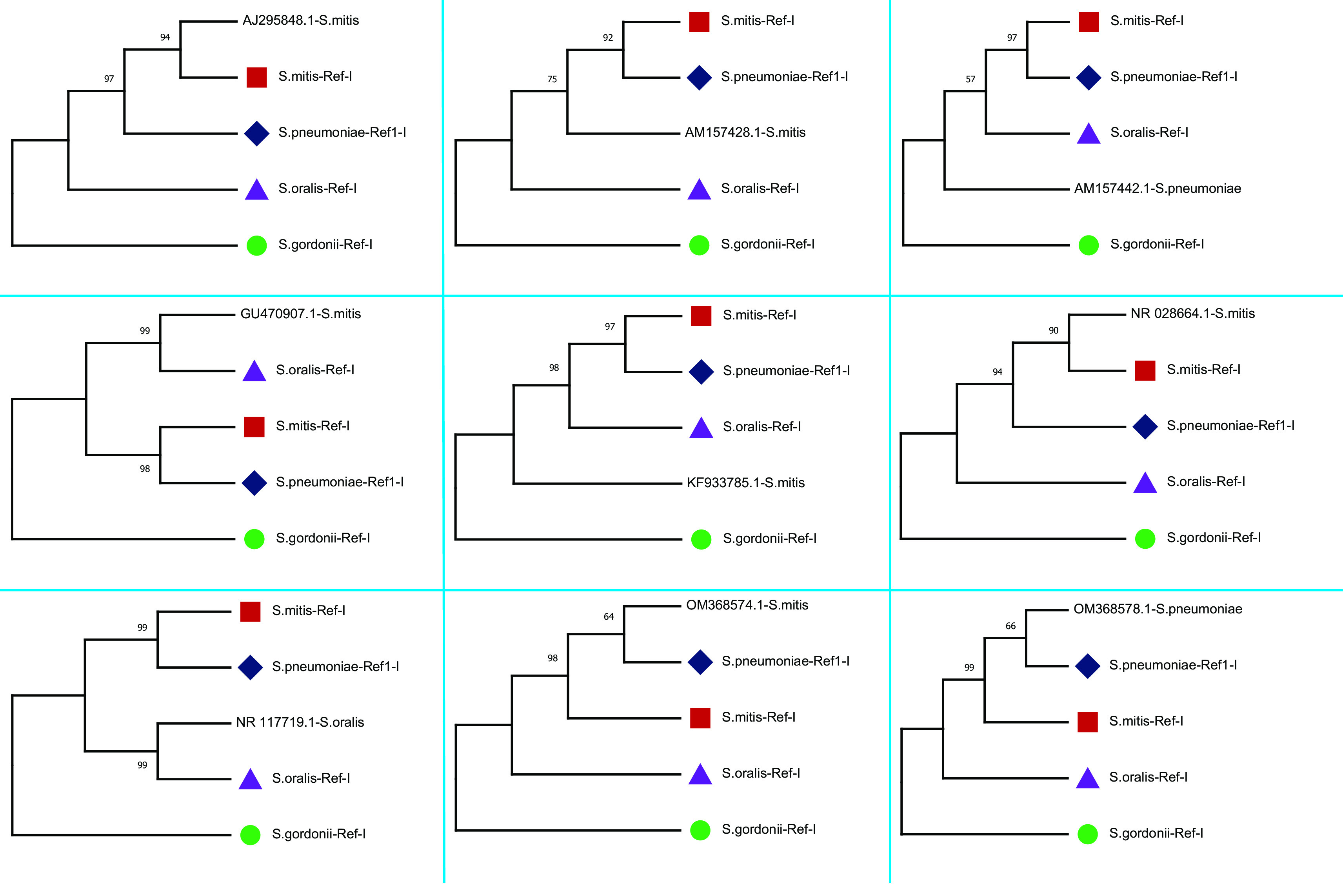
Phylogenetic tree constructed using concatenated 16S rRNA approach. The randomly selected nine 16S rRNA sequences classified as *Streptococcus* species were compared with four species-specific reference libraries constructed. The node name highlighted in shapes ( 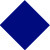, 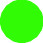, 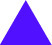, 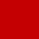) represents the four species-specific reference libraries.

The study plotted two phylogenetic tree to highlight the difference in single gene copy approach and concatenated approach. Figure 3 represent the single gene copy approach, shows phylogenetic tree of the nine 16S rRNA gene sequences selected along with the gene copies used for the construction of four concatenated species-specific reference libraries. The inclusion of misclassified sequences and intra-species variations in 16S rRNA copies may mislead the phylogenetic tree inference. Figure 4 shows the phylogenetic relationship of nine selected sequences with four concatenated species-specific reference libraries constructed. The concatenated GU470907.1 sequence showed a phylogenetic relationship with *S. oralis* and sequence OM368574.1 was genetically related to *S. pneumoniae.* Phylogenetic analysis showed that three sequences AM157428 ( *S. mitis*), KF933785 ( *S. mitis*), and AM157442 ( *S. pneumoniae*) stayed separately and might be other species than the four species tested. Furthermore, two sequences AJ295848 and NR_028664 classified as *S. mitis* showed significant similarity with concatenated 16S rRNA reference library of *S. mitis.* Similarly, sequence NR_117719 ( *S. oralis*) showed phylogenetic relationship with reference library of *S. oralis* and OM368578 ( *S. pneumoniae*) with *S. pneumoniae* reference library. These results further confirm that species-specific concatenated 16S rRNA reference libraries provide much better taxonomic resolution. Therefore, this study recommends concatenated sequences of 16S rRNA genes for sequence similarity- and phylogeny-based species identification.

## Discussion

Sequencing and analysis of the 16S rRNA encoding region is a conventional and robust method for identifying and classifying bacterial species. The barcode gene is widely used in sequence similarity, phylogeny, and metagenome-based species identification. However, the accuracy of bacterial taxonomy based on 16S rRNA barcode regions is limited by the intra-genomic heterogeneity of multiple 16S rRNA gene copies and significant sequence identity of this gene among closely related taxa. Furthermore, identification of closely related species using sequences of the 16S rRNA gene is a challenge, and it may lead to species misidentification ( Boudewijns *et al*. 2006; Church *et al*. 2020). About 15% of the bacterial genomes have only a single copy of the 16S rRNA gene, and only a minority of bacterial genomes contain identical 16S rRNA gene copies ( Vetrovsky and Baldrian 2013). The 16S rRNA gene copies can vary from 1 to 15 in a genome, and the copy number is taxon specific ( Vetrovsky and Baldrian 2013). Sequence diversity increases with the increasing 16S rRNA copy numbers. The 16S rRNA sequence variation can even be found at intra-genomic level or in different strains of a species. Amplification of a limited number of variable regions cannot achieve the same taxonomic resolution as that of the entire gene ( Johnson *et al*. 2019). Usage of misclassified 16S rRNA sequences as a reference and inappropriate bioinformatics workflows can also mislead the taxonomic assignment. To overcome these challenges, it is important to translate high throughput microbial genomic data into meaningful, actionable information that clinicians can readily understand and quickly implement for bacterial identification. Hence, the study intended to develop a species-specific catalogue of concatenated 16S rRNA gene copies that can yield better inference in sequence similarity and phylogenetic analysis.

Several bioinformatics resources are extensively used for the 16S rRNA sequence analysis and bacterial identification. However, several researchers report the sequence similarity derived through a local alignment algorithm. Earlier reports have suggested that the species belonging to the taxa Gammaproteobacteria show higher intra-species variability ( Vetrovsky and Baldrian 2013). Hence, the study estimated the percent identity of intra-genomic 16S rRNA gene copies of *E. asburiae* using local and global alignment algorithms. The reference genome of *E. asburiae* has eight 16S rRNA gene copies in its genome. The BLAST and Clustal sequence alignment algorithms yielded marginally varying results for the intra-genomic 16S rRNA gene copies. Local alignment algorithms may not consider base mismatches at the ends of sequences when calculating percent identity, while global alignment algorithms consider entire sequences. Therefore, global sequence alignment is best for estimating intra and inter-species identity for single gene copies. However, BLAST can calculate the total alignment score with multiple paralogue regions. Hence, web-based BLAST2 is suggested for estimating the sequence similarity using concatenated barcode reference libraries.

The GenBank ( Leray *et al*. 2019) and NCBI 16S RefSeq databases for bacteria ( Winand *et al*. 2020) are reliable for species-level identification and classification. However, few earlier studies have highlighted the misclassification of species and genome assemblies in public genetic databases ( Parks *et al*. 2018; Varghese *et al*. 2015). For example, the 16S rRNA sequence accession number (Ac. No.) LT707617.1 shows the organism as *Streptococcus mitis.* Conventional BLAST-based sequence similarity search shows the highest identity of 99.60% with *S. mitis* 16S rRNA sequence (Ac. No. AB002520.1). However, the 16S rRNA sequence (Ac. No. LT707617.1) did not show significant similarity with other 16S rRNA reference sequences available for *S. mitis.* Furthermore, the sequence also shows 99.44% identity with reference 16S rRNA sequences of *S. gordonii.* Hence, the study performed a sequence alignment of the sequence (Acc. No. LT707617.1) against species-specific concatenated 16S rRNA reference libraries for *S. gordonii* ( *S.gordonii-*Ref-I), and *S. mitis* ( *S.mitis-*Ref-I). The alignment resulted in a significant identity of 99.44% with *S.gordonii-*Ref-I (2279 maximum and 9041 total alignment score) than *S.mitis*-Ref-I (97.13% identity with 2119 maximum and 8449 total alignment score). Single copy BLAST results may show only a minor fraction of the difference in percent identity and maximum or total alignment score for closely related species. However, sequence similarity estimation using species-specific concatenated reference libraries shows marginal difference in total alignment score, as it is aligned against four copies. Hence, 16S rRNA analysis with a species-specific concatenated barcode reference library will give better accuracy for bacterial classification than approaches using a single copy.

Several 16S rRNA sequences show 100% identity with multiple species, which is the major challenge in sequence-based species identification. For example, the 16S rRNA sequence from *S. mitis* (Accession. No. GU470907.1; 1522 bp) shares 100% identity with the 16S rRNA gene from *S. oralis* strain ATCC 35037 genome (Ac. No. CP034442.1). Hence, the sequence (GU470907.1) aligned against the species-specific concatenated reference libraries for *S. oralis* ( *S.oralis-*Ref-I), and *S. mitis* ( *S.mitis-*Ref-I). The result showed 100% identity with *S. oralis* (2787 maximum and 10936 total alignment score), and 99.14% identity with *S. mitis* (2715 maximum and 10796 total alignment score). Further, a phylogenetic tree of GU470907.1 (1509 × 4 = 6036 bp) with reference libraries *S.mitis-*Ref-I, and *S.oralis-*Ref-I was plotted. The UPGMA-based phylogenetic tree showed that the *S. mitis* (GU470907.1) sequence is more closely related to *S. oralis* than *S. mitis* ( Figure 4). Concatenated 16S rRNA-based estimation of sequence similarity and a phylogenetic inference provides better resolution than single-gene approaches. These results show that the concatenated 16S rRNA approach is very effective in discriminating genetically closely related bacterial species. Furthermore, other studies have also highlighted that the phylogenetic tree inferred from vertically inherited protein sequence concatenation provided higher resolution than those obtained from a single copy ( Ciccarelli *et al*. 2006; Thiergart *et al*. 2014).

Recent phylogenetic studies using concatenated multi-gene sequence data highlighted the importance of incorporating variations in gene histories, which will improve the traditional phylogenetic inferences ( Devulder *et al*. 2005; Johnston *et al*. 2019). Furthermore, a single type of analysis should not be relied upon, instead, and to a certain extent, integrated bioinformatics approaches can avoid misclassification. As a cost-effective approach, the study combined substantial variations in 16S rRNA gene copies from a species to examine the performance of the single gene concatenation approach. Analyses using a concatenated 16S rRNA gene approach have the following advantages: (i) the gene is present in all the bacterial species, (ii) the gene is weakly affected by horizontal gene transfer and mutation, (iii) the approach is very cost-effective, (iv) there is a large volume of reference genomic data available for several bacterial species, (v) it is effective in discriminating closely related bacterial species, (vi) the analyses can be performed in a computer with minimum configuration, and (vii) the analyses can be employed with available tools for sequence similarity and molecular phylogeny.

## Conclusions

The concatenated 16S rRNA analyses showed that:

• Full-length 16S rRNA gene amplification provides better accuracy than inference based on partial gene sequences with a limited number of variable regions.
• Full-length 16S rRNA gene copies from whole-genome assemblies (in 'complete' stage) should be used rather than partial sequences available from the public genetic databases to construct species-specific concatenated 16S rRNA libraries and further downstream analysis.
• To avoid mismatches in the sequence alignment, trim the bases beyond the primer ends and correct the base-call errors prior to the analysis.
• Estimation of mean 16S rRNA identity at the intra-species level helps to classify the species having a higher degree of intra-genomic 16S rRNA heterogeneity.
• Four distinct 16S rRNA gene copies cover all the Parsim-Info variable sites and these can be used to construct a concatenated species-specific reference library.
• The total alignment score can be considered if the query sequence shows more or less the same percent identity with multiple species.
• It is not prudent to rely only on sequence similarity; the final decision must be based on the phylogenetic inference.
• Species-specific concatenated 16S rRNA gene libraries are recommended for sequence similarity and phylogenetic analysis.
• The limitation of the approach is that developing a species-specific reference library requires 16S rRNA copies from at least four whole genome assemblies.

## Data Availability

Zenodo: Underlying data for ‘Concatenated 16S rRNA sequence analysis improves bacterial taxonomy’, https://doi.org/10.5281/zenodo.7758747 ( Paul 2022). This project contains the following underlying data:
• Supplementary data 1: The 16S rRNA copies retrieved from the whole genome of *Enterobacter asburiae* strain ATCC 35953. • Supplementary data 2: Full-length 16S rRNA gene copies retrieved from 16 genome assemblies belonging to four *Streptococcus* species ( *S. gordonii*, *S. mitis*, *S. oralis*, and *S. pneumoniae*). • Supplementary data 3: Species-specific concatenated 16S rRNA libraries constructed for four *Streptococcus* species ( *S. gordonii*, *S. mitis*, *S. oralis*, and *S. pneumoniae*). • Supplementary data 4: Intra-species Parsim-info variable sites in the 16S rRNA gene from for four *Streptococcus* species ( *S. gordonii*, *S. mitis*, *S. oralis*, and *S. pneumoniae*). Data are available under the terms of the Creative Commons Attribution 4.0 International license (CC-BY 4.0) GenBank: *Streptococcus gordonii* strain FDAARGOS 1454 chromosome, complete genome. Accession number CP077224.1. https://www.ncbi.nlm.nih.gov/nuccore/CP077224.1 GenBank: *Streptococcus gordonii* strain NCTC7869, chromosome 1, complete genome. Accession number LR134291.1. https://www.ncbi.nlm.nih.gov/nuccore/LR134291.1 GenBank: *Streptococcus gordonii* strain KCOM 1506 (=ChDC B679), complete genome. Accession number CP012648.1. https://www.ncbi.nlm.nih.gov/nuccore/CP012648.1 GenBank: *Streptococcus gordonii* strain NCTC9124, chromosome 1, complete genome. Accession number LR594041.1. https://www.ncbi.nlm.nih.gov/nuccore/LR594041.1 GenBank: *Streptococcus mitis* B6, complete genome. Accession number NC_013853.1. https://www.ncbi.nlm.nih.gov/nuccore/NC_013853.1 GenBank: *Streptococcus mitis* strain KCOM 1350 (= ChDC B183), complete genome. Accession number CP012646.1. https://www.ncbi.nlm.nih.gov/nuccore/CP012646.1 GenBank: *Streptococcus mitis* strain SVGS_061 chromosome, complete genome. Accession number CP014326.1. https://www.ncbi.nlm.nih.gov/nuccore/CP014326.1 GenBank: *Streptococcus mitis* NCTC 12261 chromosome, complete genome. Accession number CP028414.1. https://www.ncbi.nlm.nih.gov/nuccore/CP028414.1 GenBank: *Streptococcus oralis* strain NCTC11427, chromosome 1, complete genome. Accession number LR134336.1. https://www.ncbi.nlm.nih.gov/nuccore/LR134336.1 GenBank: *Streptococcus oralis* strain 34 chromosome, complete genome. Accession number CP079724.1. https://www.ncbi.nlm.nih.gov/nuccore/CP079724.1 GenBank: *Streptococcus oralis* strain FDAARGOS_886 chromosome, complete genome. Accession number CP065706.1. https://www.ncbi.nlm.nih.gov/nuccore/CP065706.1 GenBank: *Streptococcus oralis* subsp. *dentisani* strain F0392 chromosome, complete genome. Accession number CP034442.1. https://www.ncbi.nlm.nih.gov/nuccore/CP034442.1 GenBank: *Streptococcus pneumoniae* strain 475 chromosome, complete genome. Accession number CP046355.1. https://www.ncbi.nlm.nih.gov/nuccore/CP046355.1 GenBank: *Streptococcus pneumoniae* NU83127 DNA, complete genome. Accession number AP018936.1. https://www.ncbi.nlm.nih.gov/nuccore/AP018936.1 GenBank: *Streptococcus pneumoniae* NCTC7465, chromosome 1, complete genome. Accession number LN831051.1. https://www.ncbi.nlm.nih.gov/nuccore/LN831051.1 GenBank: *Streptococcus pneumoniae* strain 6A-10 chromosome, complete genome. Accession number CP053210.1. https://www.ncbi.nlm.nih.gov/nuccore/CP053210.1 GenBank: *Streptococcus mitis* strain 127R, partial 16S rRNA gene. Accession number AJ295848.1. https://www.ncbi.nlm.nih.gov/nuccore/AJ295848.1 GenBank: *Streptococcus mitis* clone 2C4, 16S rRNA gene. Accession number AM157428.1. https://www.ncbi.nlm.nih.gov/nuccore/AM157428.1 GenBank: *Streptococcus mitis* strain NS51, partial 16S rRNA gene. Accession number NR_028664.1. https://www.ncbi.nlm.nih.gov/nuccore/NR_028664.1 GenBank: *Streptococcus mitis* bv. 2 strain F0392, partial 16S rRNA gene. Accession number GU470907.1. https://www.ncbi.nlm.nih.gov/nuccore/GU470907.1 GenBank: *Streptococcus mitis* strain ChDC B553, partial 16S rRNA gene. Accession number KF933785. https://www.ncbi.nlm.nih.gov/nuccore/KF933785.1 GenBank: *Streptococcus mitis* strain FC6528, partial 16S rRNA gene. Accession number OM368574.1. https://www.ncbi.nlm.nih.gov/nuccore/OM368574.1 GenBank: *Streptococcus pneumoniae* strain FC6532, partial 16S rRNA gene. Accession number OM368578.1. https://www.ncbi.nlm.nih.gov/nuccore/OM368578.1 GenBank: *Streptococcus pneumoniae* clone 4V4, 16S rRNA gene. Accession number AM157442. https://www.ncbi.nlm.nih.gov/nuccore/AM157442.1 GenBank: *Streptococcus oralis* subsp. *dentisani* strain 7747, partial 16S rRNA gene. Accession number NR_117719. https://www.ncbi.nlm.nih.gov/nuccore/NR_117719.1 GenBank: *Enterobacter asburiae* strain ATCC 35953 chromosome, complete genome. Accession number NZ_CP011863. https://www.ncbi.nlm.nih.gov/nuccore/NZ_CP011863.1 GenBank: *Streptococcus mitis* strain HAC11, isolate #11, partial 16S rRNA gene. Accession number LT707617. https://www.ncbi.nlm.nih.gov/nuccore/LT707617.1 GenBank: *Streptococcus mitis* strain NCTC 3165, MAFF 911479, 16S rRNA gene. Accession number AB002520.1. https://www.ncbi.nlm.nih.gov/nuccore/AB002520.1

## References

[ref1] Alachiotis N Vogiatzi E Pavlidis P : Chromatogate: a tool for detecting base mis-calls in multiple sequence alignments by semi-automatic chromatogram inspection. *Comput. Struct. Biotechnol. J.* 2013;6:e201303001. 10.5936/csbj.201303001 24688709 PMC3962156

[ref2] Altschul SF Gish W Miller W : Basic local alignment search tool. *J. Mol. Biol.* 1990;215:403–410. 10.1016/S0022-2836(05)80360-2 2231712

[ref3] Bagheri H Severin AJ Rajan H : Detecting and correcting misclassified sequences in the large-scale public databases. *Bioinformatics.* 2020;36:4699–4705. 10.1093/bioinformatics/btaa586 32579213 PMC7821992

[ref4] Baltrus DA : Divorcing strain classification from species names. *Trends Microbiol.* 2016;24:431–439. 10.1016/j.tim.2016.02.004 26947794

[ref5] Benitez-Paez A Sanz Y : Multi-locus and long amplicon sequencing approach to study microbial diversity at species level using the MinIONTM portable Nanopore sequencer. *Gigascience.* 2017;6:1–12. 10.1093/gigascience/gix043 28605506 PMC5534310

[ref6] Boudewijns M Bakkers JM Sturm PDJ : 16S rRNA gene sequencing and the routine clinical microbiology laboratory: A perfect marriage? *J. Clin. Microbiol.* 2006;44:3469–3470. 10.1128/JCM.01017-06 16954306 PMC1594676

[ref7] Caporaso JG Lauber CL Walters WA : Global patterns of 16S rRNA diversity at a depth of millions of sequences per sample. *Proc. Natl. Acad. Sci. U S A.* 2011;108:4516–4522. 10.1073/pnas.1000080107 20534432 PMC3063599

[ref8] Chakravorty S Helb D Burday M : A detailed analysis of 16S ribosomal RNA gene segments for the diagnosis of pathogenic bacteria. *J. Microbiol. Methods.* 2007;69:330–339. 10.1016/j.mimet.2007.02.005 17391789 PMC2562909

[ref9] Church DL Cerutti L Gürtler A : Performance and application of 16S rRNA gene cycle sequencing for routine identification of bacteria in the clinical microbiology laboratory. *Clin. Microbiol. Rev.* 2020;33:e00053–e00019. 10.1128/CMR.00053-19 32907806 PMC7484979

[ref10] Ciccarelli FD Doerks T Mering C von : Toward automatic reconstruction of a highly resolved tree of life. *Science.* 2006;311:1283–1287. 10.1126/science.1123061 16513982

[ref11] Clarridge JE : Impact of 16S rRNA gene sequence analysis for identification of bacteria on clinical microbiology and infectious diseases. *Clin. Microbiol. Rev.* 2004;17:840–862. 10.1128/CMR.17.4.840-862.2004 15489351 PMC523561

[ref12] Deurenberg RH Bathoorn E Chlebowicz MA : Application of next generation sequencing in clinical microbiology and infection prevention. *J. Biotechnol.* 2017;243:16–24. 10.1016/j.jbiotec.2016.12.022 28042011

[ref13] Devanga-Ragupathi NK Muthuirulandi SDP Inbanathan FY : Accurate differentiation of *Escherichia coli* and *Shigella* serogroups: challenges and strategies. *New Microbes New Infect.* 2018;21:58–62. 10.1016/j.nmni.2017.09.003 29204286 PMC5711669

[ref14] Devulder G Montclos MP de Flandrois JP : A multigene approach to phylogenetic analysis using the genus *Mycobacterium* as a model. *Int. J. Syst. Evol. Microbiol.* 2005;55:293–302. 10.1099/ijs.0.63222-0 15653890

[ref15] Ibal JC Pham HQ Park CE : Information about variations in multiple copies of bacterial 16S rRNA genes may aid in species identification. *PLoS One.* 2019;14:e0212090. 10.1371/journal.pone.0212090 30768621 PMC6377111

[ref16] Janda JM Abbott SL : 16S rRNA gene sequencing for bacterial identification in the diagnostic laboratory: Pluses, perils, and pitfalls. *J. Clin. Microbiol.* 2007;45:2761–2764. 10.1128/JCM.01228-07 17626177 PMC2045242

[ref17] Johnson JS Spakowicz DJ Hong BY : Evaluation of 16S rRNA gene sequencing for species and strain-level microbiome analysis. *Nat. Commun.* 2019;10:5011–5029. 10.1038/s41467-019-13036-1 31695033 PMC6834636

[ref18] Johnston PR Quijada L Smith CA : A multigene phylogeny toward a new phylogenetic classification of *Leotiomycetes.* *IMA Fungus.* 2019;10:1. 10.1186/s43008-019-0002-x 32647610 PMC7325659

[ref19] Kerkhof LJ Dillon KP Haggblom MM : Profiling bacterial communities by MinION sequencing of ribosomal operons. *Microbiome.* 2017;5:116. 10.1186/s40168-017-0336-9 28911333 PMC5599880

[ref20] Kumar S Stecher G Li M : MEGA X: Molecular evolutionary genetics analysis across computing platforms. *Mol. Biol. Evol.* 2018;35:1547–1549. 10.1093/molbev/msy096 29722887 PMC5967553

[ref21] Lal D Verma M Lal R : Exploring internal features of 16S rRNA gene for identification of clinically relevant species of the genus *Streptococcus.* *Ann. Clin. Microbiol. Antimicrob.* 2011;10:28. 10.1186/1476-0711-10-28 21702978 PMC3151204

[ref22] Leray M Knowlton N Ho SL : GenBank is a reliable resource for 21 ^st^ century biodiversity research. *Proc. Natl. Acad. Sci. USA.* 2019;116:22651–22656. 10.1073/pnas.1911714116 31636175 PMC6842603

[ref23] Liu Y Lai Q Shao Z : Genome analysis-based reclassification of *Bacillus weihenstephanensis* as a later heterotypic synonym of *Bacillus mycoides.* *Int. J. Syst. Evol. Microbiol.* 2018;68:106–112. 10.1099/ijsem.0.002466 29095136

[ref24] Martínez-Romero E Rodríguez-Medina N Beltrán-Rojel M : Genome misclassification of *Klebsiella variicola* and *Klebsiella quasipneumoniae* isolated from plants, animals and humans. *Salud Publica Mex.* 2018;60:56–62. 10.21149/8149 29689657

[ref25] Mateo-Estrada V Grana-Miraglia L Lopez-Leal G : Phylogenomics reveals clear cases of misclassification and genus-wide phylogenetic markers for *Acinetobacter.* *Genome Biol. Evol.* 2019;11:2531–2541. 10.1093/gbe/evz178 31406982 PMC6740150

[ref26] Parks DH Waite DW Skarshewski A : A standardized bacterial taxonomy based on genome phylogeny substantially revises the tree of life. *Nat. Biotechnol.* 2018;36:996–1004. 10.1038/nbt.4229 30148503

[ref27] Paul B Dixi G Murali TS : Genome-based taxonomic classification. *Genome.* 2019;62:45–52. 10.1139/gen-2018-0072 30649978

[ref28] Paul B : Concatenated 16S rRNA sequence analysis improves bacterial taxonomy. 2022. 10.51281/zenodo.7384709 PMC10521043 37767069

[ref29] Peker N Garcia-Croes S Dijkhuizen B : A comparison of three different bioinformatics analyses of the 16S-23S rRNA encoding region for bacterial identification. *Front. Microbiol.* 2019;10:620. 10.3389/fmicb.2019.00620 31040829 PMC6476902

[ref30] Quast C Pruesse E Yilmaz P : The SILVA ribosomal RNA gene database project: improved data processing and web-based tools. *Nucleic Acids Res.* 2013;41:D590–D596. 10.1093/nar/gks1219 23193283 PMC3531112

[ref31] Reller LB Weinstein MP Petti CA : Detection and identification of microorganisms by gene amplification and sequencing. *Clin. Infect. Dis.* 2007;44:1108–1114. 10.1086/512818 17366460

[ref32] Sabat AJ Zanten E van Akkerboom V : Targeted next-generation sequencing of the 16S-23S rRNA region for culture-independent bacterial identification increased discrimination of closely related species. *Sci. Rep.* 2017;7:1–12. 10.1038/s41598-017-03458-6 28611406 PMC5469791

[ref33] Schloss PD : Reintroducing mothur: 10 Years Later. *Appl. Environ. Microbiol.* 2020;86:e02343–e02319. 10.1128/AEM.02343-19 31704678 PMC6952234

[ref34] Sievers F Wilm A Dineen D : Fast, scalable generation of high-quality protein multiple sequence alignments using Clustal Omega. *Mol. Syst. Biol.* 2011;7:539. 10.1038/msb.2011.75 21988835 PMC3261699

[ref35] Srinivasan R Karaoz U Volegova M : Use of 16S rRNA gene for identification of a broad range of clinically relevant bacterial pathogens. *PLoS One.* 2015;10:e0117617. 10.1371/journal.pone.0117617 25658760 PMC4319838

[ref36] Stackebrandt E Mondotte JA Fazio LL : Authors need to be prudent when assigning names to microbial isolates. *Arch. Microbiol.* 2021;203:5845–5848. 10.1007/s00203-021-02599-7 34709418

[ref37] Starke R Pylro VS Morais DK : 16S rRNA gene copy number normalization does not provide more reliable conclusions in metataxonomic surveys. *Microb. Ecol.* 2021;81:535–539. 10.1007/s00248-020-01586-7 32862246 PMC7835310

[ref38] Steven B Hesse C Soghigian J : Simulated rRNA/DNA ratios show potential to misclassify active populations as dormant. *Appl. Environ. Microbiol.* 2017;83:e00696–e00617. 10.1128/AEM.00696-17 28363969 PMC5440720

[ref39] Thiergart T Landan G Martin WF : Concatenated alignments and the case of the disappearing tree. *BMC Evol. Biol.* 2014;14:212–266. 10.1186/s12862-014-0266-0 25547755 PMC4302582

[ref40] Varghese NJ Mukherjee S Ivanova N : Microbial species delineation using whole genome sequences. *Nucleic Acids Res.* 2015;43:6761–6771. 10.1093/nar/gkv657 26150420 PMC4538840

[ref41] Vetrovsky T Baldrian P : The variability of the 16S rRNA gene in bacterial genomes and its consequences for bacterial community analyses. *PLoS One.* 2013;8:e57923. 10.1371/journal.pone.0057923 23460914 PMC3583900

[ref42] Weisburg WG Barns SM Pelletier DA : 16S ribosomal DNA amplification for phylogenetic study. *J. Bacteriol.* 1991;173:697–703. 10.1128/jb.173.2.697-703.1991 1987160 PMC207061

[ref43] Winand R Bogaerts B Hoffman S : Targeting the 16S rRNA gene for bacterial identification in complex mixed samples: Comparative evaluation of second (Illumina) and third (Oxford Nanopore technologies) generation sequencing technologies. *Int. J. Mol. Sci.* 2020;21:298. 10.3390/ijms21010298 31906254 PMC6982111

[ref44] Yoon SH Ha SM Kwon S : Introducing EzBioCloud: a taxonomically united database of 16S rRNA gene sequences and whole-genome assemblies. *Int. J. Syst. Evol. Microbiol.* 2017;67:1613–1617. 10.1099/ijsem.0.001755 28005526 PMC5563544

