# Supplementary material for: Concatenated 16S rRNA sequence analysis improves bacterial taxonomy
Source: F1000Res. 2023 Sep 1;11:1530. Originally published 2022 Dec 19. [Version 3] doi: 10.12688/f1000research.128320.3 (PMC10521043; doi:10.12688/f1000research.128320.3)
Supplement: Peer review history

# Reviewer report: Reviewer response for version 3

**DOI:** 10.5256/f1000research.155249.r203269

**Published:** 2023-09-08

**Authors:** Siddaramappa Shivakumara

**Affiliations:** 1 Institute of Bioinformatics and Applied Biotechnology, Bengaluru, Karnataka, India

The revised version [version 3] addressed the minor concerns and is further improved. I approve the same for indexing.

Is the work clearly and accurately presented and does it cite the current literature?

Partly

If applicable, is the statistical analysis and its interpretation appropriate?

Not applicable

Are all the source data underlying the results available to ensure full reproducibility?

Yes

Is the study design appropriate and is the work technically sound?

Yes

Are the conclusions drawn adequately supported by the results?

Partly

Are sufficient details of methods and analysis provided to allow replication by others?

Yes

Reviewer Expertise:

Bacterial Genomics, Bacterial Taxonomy, Comparative Genomics, Pathogenomics

I confirm that I have read this submission and believe that I have an appropriate level of expertise to confirm that it is of an acceptable scientific standard.

# Reviewer report: Reviewer response for version 3

**DOI:** 10.5256/f1000research.155249.r203270

**Published:** 2023-09-08

**Authors:** Wellyzar Sjamsuridzal

**Affiliations:** 1 Department of Biology, Faculty of Mathematics and Natural Sciences, Universitas Indonesia, Depok, West Java, Indonesia

Dear Authors,

 I have read the revised version 3 of the manuscript and I found all of my concerns have been responded to. Therefore, I approve this version for indexing.

 Thank you.

 Wellyzar S.

Is the work clearly and accurately presented and does it cite the current literature?

Yes

If applicable, is the statistical analysis and its interpretation appropriate?

Yes

Are all the source data underlying the results available to ensure full reproducibility?

Yes

Is the study design appropriate and is the work technically sound?

Yes

Are the conclusions drawn adequately supported by the results?

Partly

Are sufficient details of methods and analysis provided to allow replication by others?

Yes

Reviewer Expertise:

Microbial Systematics

I confirm that I have read this submission and believe that I have an appropriate level of expertise to confirm that it is of an acceptable scientific standard.

# Reviewer report: Reviewer response for version 2

**DOI:** 10.5256/f1000research.144651.r189677

**Published:** 2023-08-21

**Authors:** Wellyzar Sjamsuridzal

**Affiliations:** 1 Department of Biology, Faculty of Mathematics and Natural Sciences, Universitas Indonesia, Depok, West Java, Indonesia

**Are the conclusions drawn adequately supported by the results?**

 Partly: The conclusion should elaborate more by considering the limitation of the study. Developing a species-specific reference library for all bacteria using concatenated 16S rRNA gene copy variants is a challenging task.

 Here are a few considerations regarding its feasibility for species identification:

 Concatenating 16S rRNA gene copy variants for sequence similarity estimation and phylogenetic inference can offer improved resolution compared to single-gene approaches. However, careful design, data interpretation, and consideration of alternative approaches are still required to account for potential biases and supplementary analyses when necessary.

1. The 16S rRNA gene is commonly used for bacterial identification, but it may not capture the full diversity of all bacterial species. While it can be effective for many bacteria, there are certain groups that may have unique or divergent sequences not adequately represented by the 16S rRNA gene.
2. Some bacterial species can have significant intraspecies genetic variation, including variations in the 16S rRNA gene. Concatenating multiple gene copy variants can help account for this variability to a certain degree, but it may not capture all the genetic diversity within a species.
3. The 16S rRNA gene is suitable for identifying bacterial species at a broader taxonomic level. However, for more precise differentiation or identification, additional genetic markers or techniques may be required, such as whole-genome sequencing or multilocus sequence typing.
4. Concatenating multiple gene copy variants from a large number of bacteria would result in a substantial increase in the complexity and size of the reference library. Managing and interpreting such a comprehensive library would require significant computational resources and expertise.

In summary, while concatenating 16S rRNA gene copy variants can be a valuable approach for bacterial identification, developing a species-specific reference library for all bacteria using this method may have limitations. It is important to consider the diversity and variability within bacterial species and explore additional techniques for more precise identification when necessary.

Is the work clearly and accurately presented and does it cite the current literature?

Yes

If applicable, is the statistical analysis and its interpretation appropriate?

Yes

Are all the source data underlying the results available to ensure full reproducibility?

Yes

Is the study design appropriate and is the work technically sound?

Yes

Are the conclusions drawn adequately supported by the results?

Partly

Are sufficient details of methods and analysis provided to allow replication by others?

Yes

Reviewer Expertise:

Microbial Systematics

I confirm that I have read this submission and believe that I have an appropriate level of expertise to confirm that it is of an acceptable scientific standard, however I have significant reservations, as outlined above.

# Response

**Published:** 2023-08-28

**Authors:** Bobby Paul

**Affiliations:** Manipal University, India

Thank you for the critical comments. I have included the limitation of this approach in the conclusion section, and the manuscript has been updated. The study used four genetically related species for the demonstration. Concatenated 16S rRNA analysis yielded better resolution than a single copy. Yes, developing concatenated species-specific 16S rRNA reference libraries for entire bacterial populations is challenging. The study developed reference libraries for four species. More than 552,575 whole-genome sequences are currently (Aug 2023) available for bacterial species in the Genome database. Hopefully, these datasets can be used for the development of species-specific reference libraries for remaining bacterial species. Further, we are in the process of developing reference libraries for other bacterial species and hosting a web server for easy analysis.

 Here is the response to the specific queries.

1. The gene encoding the 16S rRNA is ~1500 base pair (bp) long, present in all bacterial populations, and the gene consists of nine variable regions. In sequence analysis, the variable regions are differentiating species. Hence, the proposed approach is applicable to delineate all the bacterial populations. However, as it is ~1500 bp, sequencing of this gene is not sufficient to estimate the genome-wide difference. Estimations such as average nucleotide identity (ANI), Genome BLAST Distance Phylogeny approach can be applied to whole genome sequences.
2. Genetic differences can be found between strains within a species, as well as between intra-genomic 16S rRNA gene copies. The approach proposed in this manuscript is exclusively to avoid misclassification due to genetic variations in the intra-species 16S rRNA gene copies. The 16S rRNA sequence analysis is not sufficient to estimate the entire genetic difference. Several whole genome-based approaches exist for genome-wide diversity estimation and bacterial taxonomy. The 16S rRNA sequencing is considered a cost-effective, gold-standard single gene approach for species identification. Hence, the method is more appropriate for bacterial species identification. The rate of mutations and lateral gene transfer is high in bacterial populations. However, 16S rRNA is less affected by these genetic changes. Further, factors such as genome size, genetic rearrangements, and unique genomic regions can slightly affect whole genome based species identification. However, concatenated 16S rRNA analysis won't be affected by these factors.
3. Approaches to bacterial taxonomy have improved from microscopic examination to genome-based methods over the years. However, few earlier studies have highlighted the misclassification of species and genome assemblies in public genetic databases (Parks et al. 2018; Varghese et al. 2015). The manuscript explains the proof of concept. Whether concatenation of multiple copies of 16S rRNA can improve bacterial taxonomy. The study used four closely related *Streptococcus* species ( *S. gordonii*, *S. mitis, S. oralis*, and *S. pneumoniae*), which is difficult to differentiate with current genome based approaches for bacterial taxonomy. The results show the proposed concatenated 16S rRNA analysis can easily differentiate bacterial species, even sharing high genetic similarity. Although, estimations such as average nucleotide identity (ANI), genome-to-genome distance calculation, Genome BLAST Distance Phylogeny approach can be applied to whole genome sequences.
4. Thank you. The study was completed using a laptop that has a 4GB of RAM. Whole genome sequence data available in the public genetic databases can be used to develop reference libraries for remaining bacterial species. The analyses can be employed with currently available tools for sequence similarity and molecular phylogeny. Therefore, interpreting results should not pose a challenge for researchers. However, we are in the process of hosting a web server for more easy and appropriate analysis of global bacterial populations.

Kindly respond if you have any other queries or concerns.

 Kind Regards

 Bobby Paul

# Reviewer report: Reviewer response for version 2

**DOI:** 10.5256/f1000research.144651.r168593

**Published:** 2023-04-11

**Authors:** Siddaramappa Shivakumara

**Affiliations:** 1 Institute of Bioinformatics and Applied Biotechnology, Bengaluru, Karnataka, India

The author has addressed most of my technical and non-technical (e.g., writing style and presentation) concerns. Version 2 is considerably improved compared to Version 1 in terms of readability, flow, and impact. However, I still believe there is scope to further improve the Discussion section. Nevertheless, I approve Version 2 without any further reservations.

Is the work clearly and accurately presented and does it cite the current literature?

Partly

If applicable, is the statistical analysis and its interpretation appropriate?

Not applicable

Are all the source data underlying the results available to ensure full reproducibility?

Yes

Is the study design appropriate and is the work technically sound?

Yes

Are the conclusions drawn adequately supported by the results?

Partly

Are sufficient details of methods and analysis provided to allow replication by others?

Yes

Reviewer Expertise:

Bacterial Genomics, Bacterial Taxonomy, Comparative Genomics, Pathogenomics

I confirm that I have read this submission and believe that I have an appropriate level of expertise to confirm that it is of an acceptable scientific standard.

# Reviewer report: Reviewer response for version 1

**DOI:** 10.5256/f1000research.140896.r158444

**Published:** 2023-02-10

**Authors:** Siddaramappa Shivakumara

**Affiliations:** 1 Institute of Bioinformatics and Applied Biotechnology, Bengaluru, Karnataka, India

The manuscript entitled “Concatenated 16S rRNA sequence analysis improves bacterial taxonomy [version 1]” by Bobby Paul is generally well written and reports interesting results. The methods are appropriate and the analyses meet the quality standards.

 However, there are some concerns about the clarity and presentation of results. Major revisions in the text are required to improve clarity and comprehensibility. My suggestions/critiques for revising the text in different sections of the manuscript are provided. Author’s attention has also been drawn to technical concerns, which need to be carefully addressed.

 The Discussion section is rather lengthy; it should be shortened and include only relevant results/studies (present and previous). I would be happy to provide detailed critiques on the Discussion section after the manuscript goes through the first round of revision.

*Is the work clearly and accurately presented and does it cite the current literature?*

 Some statements/results are missing citations and references. These have been highlighted and need to be revised.

*Are the conclusions drawn adequately supported by the results?*

 Some conclusions are unsubstantiated, and require relevant data and proper interpretation. These have been highlighted and need to be revised.

**Introduction**

 "The 16S ribosomal RNA (16S rRNA) encoding region is extensively studied to identify and classify bacterial species. The 16S rRNA is a conserved component of the 30S small subunit of a prokaryotic ribosome"

**Please revise this as**

 "The DNA region encoding the 16S ribosomal RNA (16S rRNA)  is extensively studied, and used to identify and classify bacterial species. The 16S rRNA is a conserved component of the small subunit (30S) of the prokaryotic ribosome".

 The gene is ~1500 base pair (bp) long, and it consists of nine variable regions.

**Please revise this as**

 "The gene encoding the 16S rRNA is base pair (bp) long, and it consists of nine variable regions"

 "For decades, the sequence of the 16S rRNA gene has been used as a potential molecular marker in culture-independent methods to identify and classify diverse bacterial communities ( Clarridge, 2004; Johnson *et al*. 2019)"

**Please revise this as**

 "The sequence of the 16S rRNA gene has been extensively used as a molecular marker in culture-independent methods to identify and classify diverse bacterial communities"

 "The 16S rRNA sequences are currently being used as an accurate and rapid method to study bacterial evolution, phylogenetic relationships, populations in an environment, and quantification of abundant taxa"

**Please revise this as**

 "Bacterial 16S rRNA sequences are currently being used to study the evolution, phylogenetic relationships, and environmental abundanceof various taxa".

 "Despite the wide range of applications, a few shortcomings limit the accuracy of results derived through the 16S rRNA sequence analysis. One such aspect is that the 16S rRNA gene has poor discriminatory power at the species level ( Winand *et al*. 2020), and the copy number can vary from 1 to 15 or even more ( Vetrovsky and Baldrian, 2013; Winand *et al*. 2020)."

**Please revise this as**

 "Although 16S rRNA sequence analyses are the mainstay of taxonomic studies of bacteria, there are some limitations. For example, the 16S rRNA gene has poor discriminatory power at the species level ( Winand *et al*. 2020), and the copy number is highly variable ( Vetrovsky and Baldrian, 2013; Winand *et al*. 2020). ". 

 "The presence of multiple variable copies of this gene makes distinct data for a species."

**The above statement is a little confusing, and should be revised. Their presence in itself may not "make distinct data".**

 "Hence, gene copy normalization (GCN) is necessary prior to sequence analysis."

**Please revise this as**

 "Therefore, gene copy normalization (GCN) may be necessary prior to sequence analysis.

 "However, studies show that the GCN approach does not improve the 16S rRNA sequence analyses in real scenarios and suggests a comprehensive species-specific catalogue of gene copies ( Starke *et al*. 2021)".

**Please revise this as**

 "However, GCN may not improve the 16S rRNA sequence analyses in all scenarios, and comprehensive, species-specific catalogues of 16S rRNA gene copies may be necessary ( Starke *et al*. 2021).

 "Secondly, the intra-genomic variations between the 16S rRNA gene copies were observed in several bacterial genome assemblies ( Paul *et al*. 2019)."

**Please revise this as**

 "Furthermore, intra-species variations in the number of copies of the16S rRNA gene were observed in several bacterial genome assemblies"

 "Only a minority of the bacterial genomes harbor identical 16S rRNA gene copies, and sequence diversity increases with increasing copy numbers ( Vetrovsky and Baldrian, 2013). "

**Please revise this as**

 "Only a few bacterial species contain identical 16S rRNA gene copies, and sequence diversity increases with increasing copy numbers of 16S rRNA genes".

 "Further, currently available 16S rRNA-based bioinformatics approaches are not always amenable to classify bacterium at the species level due to high inter-species sequence similarities ( Peker *et al*. 2019; Deurenberg *et al*. 2017)."

**Please revise this as**

 "The high levels of similiarty of the 16S rRNA genes across some bacterial species poses a major challege for taxonomic studies using bioinformatics methods".

 “A few other issues are also related to the sequencing and bioinformatics analysis of 16S rRNA gene regions. These include the purity of bacterial isolates, the quality of isolated DNA, and the possibility of chimeric molecules ( Janda and Abbott, 2007; Church *et al*. 2020)”

**Please revise this as**

 Factors such as purity of bacterial cultures, quality of the purified DNA samples, and potential DNA chimeras should be carefully considered while sequencing and analysis of 16S rRNA genes.

 “Base-call errors can also mislead the sequence identity and phylogenetic inferences ( Alachiotis *et al*. 2013).

**Please revise this as**

 “Sequencing errors can lead to misidentification of bacteria and phylogenetic anomalies”.

 “The other concerns on sequence-based analysis, comparison, and species identification include the number of base ambiguities processed, gaps generated during sequence comparison, and algorithm (local or global) used for the sequence alignment.”

**Please revise this as**

 “Other concerns include sequence ambiguities, gaps generated during DNA sequencing and sequence comparisons, and choosing the appropriate algorithm (local or global) for sequence alignment.”.”

 “The local alignment algorithm is extensively used for sequence similarity-based species identification. Several studies were conducted to identify the best variable region or combination of variable regions for bacterial classification, and a consensus remains to be implemented ( Janda and Abbott, 2007; Johnson *et al*. 2019; Winand *et al*. 2020).”

**Please revise this as**

 “Since the local alignment algorithm is extensively used for sequence similarity-based comparisons, it is important to carefully consider whether a single variable region or a combination of variable regions of the 16S rRNA gene would be ideal for bacterial classification.”

 “Usage of misclassified sequence as a reference and improper bioinformatics workflows mislead the bacterial taxonomy.

**Please revise this as**

 “Using erroneous 16S rRNA sequences as references and improper bioinformatics workflows can mislead bacterial identification”. 

 "Further, the growth of bioinformatics and genetic data has placed genome-based microbial classification with researchers with little or no taxonomic experience, which may also mislead the bacterial taxonomy ( Baltrus, 2016)”.

**Kindly revise the above statement because it is too complex and confusing.**

 “A few bacterial identification systems with high resolution have been developed using the sequence of polymerase chain reaction (PCR) amplified ∼4.5 kb long 16S–23S rRNA regions (Benítez-Páez and Sanz, 2017; Sabat *et al*. 2017; Kerkhof *et al*. 2017).”

**Please revise this as**

 “Other methods for bacterial identification include the sequencing and analysis of the polymerase chain reaction (PCR) amplified ∼4.5 kb 16S–23S rRNA regions (Benítez-Páez and Sanz, 2017; Sabat *et al*. 2017; Kerkhof *et al*. 2017).”

 “However, these approaches have a few limitations, such as the lack of reference 16S–23S rRNA sequence databases and complementary bioinformatics resources for reliable species identification ( Sabat *et al*. 2017)”.

**Please revise this as**

 “However, the 16S–23S rRNA sequence-based method is less practical due to the lack of appropriate reference sequence databases and reliable tools/methods for sequence analysis”

 “The recent advancements in bioinformatics workflows ( Winand *et al*. 2020; Schloss, 2020) and reference databases such as SILVA, EzBioCloud ( Quast *et al*. 2013; Yoon, 2017) improved 16S rRNA-based bacterial taxonomy. However, a few recent genome-based studies highlighted the misclassification incidences in bacterial species and genome assemblies ( Steven *et al*. 2017; Martínez-Romero, *et al*. 2018; Mateo-Estrada *et al*. 2019; Bagheri *et al*. 2020)”.

**The first part here talks about 16S rRNA-based bacterial taxonomy, and the second part talks about genome assemblies. It is not clear what the connection is. Kindly revise these two sentences. A suggestion for revision is shown below (although even this suggestion fails to convey the connection):**

  “Although improvements in reference databases (such as SILVA and EzBioCloud) and bioinformatics workflows have facilitated 16S rRNA-based bacterial taxonomy, recent genome-based studies have indicated that incidences of misclassification of bacterial species and erroneous genome assemblies.”

 “Nowadays, conventional and high throughput sequencers can amplify all the nine variable regions of the 16S rRNA gene”.

**Please revise as**

 “The entire 16S rRNA gene (~1500 bp) can be amplified and sequenced using the conventional or high throughput sequencing methods”.

 “Although, many 16S rRNA-based bacterial identification studies lack a complete set of variable regions ( Stackebrandt *et al*. 2021)”.

**Please revise as**

 “However, many 16S rRNA sequence-based bacterial identification studies do not seem to include all of these nine variable regions ( Stackebrandt *et al*. 2021)”.

 “The classical and high throughput sequencing technologies produce a large volume of whole-genome data. There is an urgent need to translate the genomic data for convenient microbiome analyses that ensure clinical practitioners can readily understand and quickly implement it ( Church *et al*. 2020)”.

**Please revise as**

 “Due to the large volume of whole-genome data that is being produced by high throughput sequencing technologies, there is an urgent need to develop methods for analyzing this data for accurate identification of bacteria. It is also important to envisage that clinical practitioners would be able to understand these methods and implement them quickly ( Church *et al*. 2020)”.

 “Hence, the study intended to demonstrate a workflow to develop species-specific concatenated 16S rRNA reference libraries and its analysis. The species-specific libraries can yield better resolution in sequence similarity and phylogeny based bacterial classification approaches”.

**Please revise as**

 “This study aimed to develop a workflow for accurate identification of bacteria using concatenated, species-specific 16S rRNA sequences. It was hoped that the species-specific libraries would yield much better resolution in sequence similarity- and phylogeny-based bacterial classification”.

**Methods**

*Estimation of variations in intra-genomic 16S rRNA gene copies*

 “Sequence alignment of 16S rRNA copies at the intra-genomic level shows a higher degree of variability in species belonging to the *Firmicutes* and *Proteobacteria* ( Vetrovsky and Baldrian, 2013; Ibal *et al*. 2019)”.

**Please revise this as**

 “It has been reported that sequence alignment of 16S rRNA alleles at the intra-genomic level shows a higher degree of variability in species belonging to the *Firmicutes* and *Proteobacteria* ( Vetrovsky and Baldrian, 2013; Ibal *et al*. 2019)”.

 “Hence, the study used eight 16S rRNA copies (Underlying data: Supplementary data 1 ( Paul, 2022)) retrieved from the whole genome of *Enterobacter asburiae* strain ATCC 35953 (NZ_CP011863.1)”.

**Please revise this as**

 “Therefore, this study used eight 16S rRNA alleles (Underlying data: Supplementary data 1 ( Paul, 2022)) retrieved from the complete genome of *Enterobacter asburiae* strain ATCC 35953 (NZ_CP011863.1)”.

 “The BLAST+ 2.13.0 (RRID:SCR_004870; Altschul *et al*. 1990) and Clustal Omega 1.2.4 (RRID:SCR_001591; Sievers *et al*. 2011) sequence alignment algorithms were used to estimate intra-genomic variability between the 16S rRNA gene copies”.

**Please revise this as**

 “To estimate intra-genomic variability between these 16S rRNA alleles, BLAST+ 2.13.0 (RRID:SCR_004870; Altschul *et al*. 1990) and Clustal Omega 1.2.4 (RRID:SCR_001591; Sievers *et al*. 2011) sequence alignment algorithms were used”.

 “Phylogenetic relatedness between intra-genomic 16S rRNA copies were estimated using the Maximum Likelihood method (Tamura-Nei model; 500 bootstrap replicates) with MEGA software (version 11; RRID: SCR_000667; Kumar *et al*. 2018).”

**Please revise this as**

 “Phylogenetic analysis of these 16S rRNA alleles were performed using the maximum likelihood method (Tamura-Nei model; 500 bootstrap replicates) and the MEGA software (version 11; RRID: SCR_000667; Kumar *et al*. 2018)”.

*Construction of species-specific concatenated 16S rRNA reference libraries*

 “Previous studies have reported that several bacterial species share more than 99% sequence identity in the 16S rRNA encoding region”.

**Please revise this as**

 “Previous studies have reported that the genes encoding 16S rRNA from several bacterial species share >99% sequence identity”. [ALSO PROVIDE A REFERENCE HERE]

 “Hence, the 16S rRNA-based bacterial identification methods failed to discriminate such genetically related species ( Deurenberg *et al*. 2017; Devanga-Ragupathi *et al*. 2018)”.

**Please revise this as**

 “Therefore, the 16S rRNA-based methods failed to correctly identify bacterial species that are genetically closely related ( Deurenberg *et al*. 2017; Devanga-Ragupathi *et al*. 2018)”.

 “It has been reported that *Streptococcus mitis* and *Streptococcus pneumoniae* are almost indistinguishable from each other based on the sequence similarity of their 16S rRNA regions ( Reller *et al*. 2007; Lal *et al*. 2011)”.

**Please revise this as**

 It has been reported that 16S rRNA-based methods cannot **distinguish between *Streptococcus mitis* and *Streptococcus pneumoniae*  due to the high sequence similarity ( Reller *et al*. 2007; Lal *et al*. 2011).

 “To develop species-specific barcode reference libraries, the study used 16S rRNA gene copies from whole-genome assemblies of four closely related species of *Streptococcus* ( *S. gordonii*, *S. mitis*, *S. oralis,* and *S. pneumoniae*)”.

**Please DELETE the above, because it is repeated below.**

 “More than 463,000 whole-genome assemblies are currently available for prokaryotes at the Genome database (RRID:SCR_002474; https://www.ncbi.nlm.nih.gov/genome)”.

**Please revise this as**

 ‘More than 463,000 whole-genome sequences are currently (please provide a date here) available for prokaryotes (please specify if this is only for bacteria, or also includes archaea) in the Genome database (RRID:SCR_002474; https://www.ncbi.nlm.nih.gov/genome)”.

 “Most microbial genomes were sequenced with high throughput sequencing technologies such as Illumina/Ion-Torrent (short read sequencing) and PacBio/Nanopre (long read sequencing”).

**Please revise this as**

 “Many of these genomes were sequenced using high throughput sequencing technologies such as Illumina/Ion-Torrent (short read sequencing) and PacBio/Nanopre (long read sequencing)”.

 “Further, many of these whole-genome assemblies are derived through a hybrid assembly of short and long read sequence data”.

**Please revise this as**

 “Furthermore, most of these whole-genome sequences were obtained after a hybrid assembly of short and long read sequence data”.

 “The large volume of high throughput data can be effectively used to develop advanced genome-based approaches for microbial systematics”.

**Please revise this as**

 “This extensive, high throughput data can be effectively used to develop advanced genome-based methods for microbial systematics.”

 The genomic data is available in four assembly completion levels (contig, scaffold, chromosome, and complete). However, the study used only the genomes assemblies in the 'complete' stage to retrieve 16S rRNA gene copies.

**Please revise this as**

 Although the genomic data is available in four levels (contig, scaffold, chromosome, and complete), this study used only the complete genomes to retrieve 16S rRNA genes.

 “The study retrieved full-length 16S rRNA gene copies from 16 genome assemblies belonging to four *Streptococcus* species ( *S. gordonii*, *S. mitis*, *S. oralis,* and *S. pneumoniae”*).

**Please revise this as**

 “To develop species-specific barcode reference libraries, this study retrieved full-length 16S rRNA genes from 16 complete genome sequences belonging to four *Streptococcus* species ( *S. gordonii*, *S. mitis*, *S. oralis,* and *S. pneumoniae*)".

 “The detailed information on the dataset used to develop species-specific concatenated reference libraries is provided in Table 1 and the sequences are provided in the underlying data (Supplementary data 2 ( Paul, 2022))”

**Please revise this as**

 “Details of the dataset used to develop species-specific concatenated reference libraries are provided in Table 1, and the sequences are provided in the underlying data (Supplementary data 2 ( Paul, 2022))”

 “To maintain equal length, sequences were trimmed out beyond the universal primer pair fD1-5'-GAG TTT GAT CCT GGC TCA-3' and rP2-5'-ACG GCT AAC TTG TTA CGA CT-3' ( Weisburg *et al*. 1991) for full-length 16S rDNA amplification”.

**Please revise this as**

 “Sequences were trimmed beyond the universal primer pair (fD1-5'-GAG TTT GAT CCT GGC TCA-3' and rP2-5'-ACG GCT AAC TTG TTA CGA CT-3', which are used  for full-length 16S rDNA amplification, Weisburg *et al*. 1991) to maintain uniform length [PLEASE MENTION THIS IN BASE PAIRS]"

 “The study used MEGA 11 software to perform multiple sequence alignment and identify the intra-species parsimony informative (Parsim-info) variable sites”.

**Please revise this as**

 “To perform multiple sequence alignment and identify the intra-species parsimony informative (Parsim-info) variable sites, the MEGA 11 software was used”.

 “A species-specific barcode reference library covering entire Parsim-info variable sites was constructed by concatenating four 16S rRNA gene copies representing four different strains of a species”.

**Please revise this as**

 “A species-specific barcode reference library that covers the entire Parsim-info variable sites was constructed by concatenating four 16S rRNA gene copies from four different strains of a species”.

 “The rationale behind the selection of four copies for a species-specific barcode reference library is: (i) a maximum of four variations can be found on a single site, and (ii) earlier studies have shown that the mean 16S rRNA copies per genome is four ( Vetrovsky and Baldrian, 2013)”.

**Please revise this as**

 “The rationale for the selection of four copies for constructing a species-specific barcode reference library was: (i) a maximum of four variations can be found at a single site, and (ii) earlier studies have shown that the mean 16S rRNA copies per genome is four ( Vetrovsky and Baldrian, 2013)”.

*Demonstration of concatenated 16S rRNA in sequence similarity and phylogeny*

 “The study analyzed a few cases to demonstrate the classical sequence similarity and phylogenetic analysis using concatenated species-specific 16S rRNA reference libraries”.

**Please revise this as**

 “This study analyzed a few cases to demonstrate (i) the classical sequence similarity and (ii) phylogenetic analysis using concatenated species-specific 16S rRNA reference libraries”.

 “The study used nine Sanger sequenced 16S rRNA gene copies showing higher sequence similarity with multiple species of *Streptococcus* retrieved from the GenBank database (RRID:SCR_002760)”.

**Please revise this as**

 “Nine 16S rRNA gene copies (sequenced using the Sanger method) showing higher sequence similarity to the 16S rRNA genes of multiple species of *Streptococcus* were retrieved from GenBank database (RRID:SCR_002760)”.

 “The web based BLAST2 (version 2.13.0) program for aligning two or more sequences was used to estimate the maximum score, total alignment score, and sequence identity”.

**Please revise this as**

 “The web based BLAST2 (version 2.13.0) program for aligning two or more sequences was used to estimate the maximum score, total alignment score, and sequence identity of these nine 16S rRNA”.

 “A single copy of the 16S rRNA region derived through Sanger sequencing or retrieved from a whole-genome assembly can be considered as ‘Query sequence”.

**Please revise this as**

 “A single 16S rRNA gene (sequenced using the Sanger method or retrieved from a whole-genome assembly was the ‘Query sequence’”. [IT IS NOT CLEAR WHETHER THIS GENE WAS FROM *Streptococcus.* PLEAASE CLARIFY THIS]

 “The concatenated species-specific reference libraries must be provided in the ‘Subject sequence’ section”.

**Please revise this as**

 “The concatenated species-specific reference libraries were provided in the ‘Subject sequence’ window”.

 “To perform phylogenetic analysis, it is mandatory that the target sequence (length = n bp) has to be concatenated four times (length = 4 × n bp), appending next to the last base”.

**Please revise this as**

 “To perform phylogenetic analysis, it is mandatory that the target sequence (length = n bp) be concatenated four times (length = 4 × n bp)”.

 “Phylogenetic relatedness was estimated using the Maximum Likelihood method (Tamura-Nei model; 500 bootstrap replicates) with MEGA 11 software”.

**Please revise this as**

 “Phylogenetic analysis was performed as indicated above/”

**Results**

**Intra-genomic 16S rRNA variations in *Enterobacter asburiae***

 Historically, the 16S rRNA gene sequences were used to identify known and new bacterial species.

**Please revise this as**

 Historically, sequences of the 16S rRNA genes have been used to identify known and new bacterial species.

 However, this method is impacted by several factors such as amplification efficiency, poor discriminatory power at the species level, multiple polymorphic 16S rRNA gene copies, and improper bioinformatics workflows for the data analysis.

**Please revise this as**

 However, efficiency of PCR-based amplification, poor discrimination at the species level, multiple polymorphic 16S rRNA gene copies, and improper bioinformatics workflows for the data analysis can impact the identification. [PLEASE PROVIDE A REFERENCE HERE]

 The *E. asburiae* genome had eight 16S rRNA gene copies that showed a mean identity of 99.29% in sequence alignment using Clustal Omega (global alignment), whereas BLAST (local alignment) analysis resulted in an average of 99% identity between the copies ( Table 2).

**Please revise this as**

 The genome of *E. asburiae* contains eight copies of the 16S rRNA gene. Analysis using Clustal Omega (global alignment) and BLAST (local alignment) showed that the sequences of these eight alleles had average identities of 99.29 and 99%, respectively ( Table 2).

 Hence, the selection of an appropriate algorithm has a significant role in the estimation of percent identity, and a vital role in sequence-based species delineation.

**Please revise this as**

 Therefore, choosing the appropriate algorithm/tool is critical for the estimation of sequence identities and sequence-based species delineation.

 Global sequence alignment programs generally perform better for highly identical sequence pairs, and the algorithm considers all the bases for the estimation of sequence identity. The multiple sequence alignment showed 22 variable sites in 16S rRNA gene copies of the *E. asburiae* genome ( Figure 1).

**Please revise this as**

 For analyzing sequence pairs that are highly identical, global sequence alignment programs/tools seem to be more appropriate because they consider all the nucleotides for the estimation of sequence identity.

 The multiple sequence alignment showed 22 variable sites in 16S rRNA gene copies of the *E. asburiae* genome ( Figure 1).

**Please revise this as**

 Multiple sequence alignment [PLEASE MENTION THE TOOL/ALGORITHM HERE, AND ALSO IN THE LEGEND OF FIGURE 1] of the sequences of the eight alleles of the 16S rRNA gene in the genome of *E. asburiae* showed 22 variable sites ( Figure 1).

 The evolutionary relationship between species is usually represented in a phylogenetic tree drawn using a single barcode gene, multiple genes, or whole genomes.

**Please revise this as**

 The evolutionary relationship between species is usually represented using a phylogenetic tree based on the analysis of a single gene, multiple genes, or whole genomes.

 However, bacterial species nomenclature is mainly designated based on the confidence obtained from the phylogenetic tree derived through single copy 16S rRNA analysis.

**Please revise this as**

 However, bacterial identification and classification is mainly based on the phylogenetic analysis of single copies of 16S rRNA genes

 To highlight how the intra-genomic 16S rRNA variations influence the species delineation, a phylogenetic tree was constructed using eight 16S rRNA gene copies of *E. asburiae* reference genome showing multiple nodes ( Figure 2).

**Please revise this as**

 A phylogenetic tree was constructed to understand how variations in the sequences of the eight alleles of the 16S rRNA gene in the genome of *E. asburiae* influence species delineation ( Figure 2).

 The sequence similarity and phylogeny-based analysis indicate that the intra-genomic variations in 16S rRNA copies may mislead the bacterial taxonomy in single gene copy approaches.

**IT IS NOT CLEAR TO ME HOW THIS ANALYSIS “**indicate(s) that the intra-genomic variations in 16S rRNA copies may mislead the bacterial taxonomy in single gene copy approaches”. **KINDLY ELABORATE ON THE SAME.**

**Species-specific concatenated 16S rRNA libraries**

 The study selected four *Streptococcus* species ( *S. gordonii*, *S. mitis*, *S. oralis*, and *S. pneumoniae*) to construct species-specific concatenated 16S rRNA reference libraries.

**Please revise this as**

 This study selected four species of *Streptococcus* ( *S. gordonii*, *S. mitis*, *S. oralis*, and *S. pneumoniae*) to construct species-specific concatenated reference libraries based on 16S rRNA gene sequences obtained from complete genomes.

 The study used 16S rRNA copies retrieved from four whole genome assemblies in the ‘complete’ stage to construct a species-specific barcode library.

**Please DELETE the above sentence.**

 Four copies of the 16S rRNA gene are required to construct the concatenated library for a species.

**Please revise this as**

 Sequences from four copies of the 16S rRNA gene are required to construct a concatenated library for a species [PLEASE PROVIDE A REFERENCE HERE].

 The details of constructed species-specific libraries are listed in Table 1 and the sequence is provided in the underlying data (Supplementary data 3 ( Paul, 2022)).

**Please revise this as**

 The details of species-specific libraries are listed in Table 1 and the sequences are provided in the underlying data (Supplementary data 3 ( Paul, 2022)). THIS REFERENCE IS INCOMPLETE IN THE LIST, AND THE LINK IS NOT WORKING. PLEASE CHECK ANC CORRECT.

 ALSO, IN THE TITLE OF TABLE 1, “for the development of concatenated” SHOULD BE REVISED AS “for the construction of concatenated”

 The 16S rRNA sequence analysis shows 24 Parsim-info variable sites for *S. oralis*, 11 variations in *S. mitis*, seven variations in *S. gordonii,* and six variations found in *S. pneumoniae.* 

**Please revise this as**

 Analysis using the sequences of 16S rRNA genes showed 24, 11, 7, and 6 Parsim-info variable sites for *S. oralis*, *S. mitis*, *S. gordonii,* and *S. pneumoniae*, respectively. [PLEASE PROVIDE/SHOW THE DATA FOR THIS]

 The observed intra-species Parsim-info variable sites are residing on both conserved and variable regions of the 16S rRNA gene.

**Please revise this as**

 The intra-species [PLEASE CHECK IF THIS SHOULD BE “inter-species”] Parsim-info variable sites were located in both the conserved and variable regions of the 16S rRNA gene. [PLEASE PROVIDE/SHOW THE DATA FOR THIS]

 The study used full-length 16S rRNA copies from four different strains to highlight the variations at the species level.

**Please revise this as**

 This study used full-length sequences of 16S rRNA genes from four different species to check the variations at the species level.

 However, a large volume of partial 16S rRNA sequences are available in the public genetic databases.

**Please revise this as**

 However, a large number of partial sequences of 16S rRNA genes are available in the public genetic databases.

 In such cases, a species-specific concatenated 16S rRNA reference library can be developed with partial sequences.

**Please revise this as**

 In such cases, a species-specific concatenated reference library can be constructed using partial sequences.

 Intra-species variation on 16S rRNA gene copies influences the sequence based bacterial taxonomy.

**Please revise this as**

 Intra-species [PLEASE CHECK IF THIS SHOULD BE “inter-species”] variations in the sequences of 16S rRNA gene copies influences the sequence-based bacterial identification. [PLEASE PROVIDE/SHOW THE DATA FOR THIS,  OR SUBSTANTIATE THE CONCLUSION]

 Hence, the concatenated 16S rRNA approach yields better resolution than single copy analysis in classical sequence similarity and phylogeny based species identification approaches.

**Please revise this as**

 Therefore, concatenation of the sequences of 16S rRNA genes/alleles provides much better resolution compared to analysis using sequences from a single copy of the 16S rRNA gene. PLEASE NOTE: IN THE ABSENCE OF DATA, THIS STATEMENT REMAINS UNSUBSTANTIATED.

**Demonstration of concatenated 16S rRNA based species identification**

 The study compared nine 16S rRNA sequences representing *Streptococcus* species ( Table 3) with species-specific concatenated reference libraries.

**Please revise this as**

 This study compared sequences of nine 16S rRNA genes from different species of *Streptococcus* ( Table 3) using species-specific concatenated reference libraries.

 Concatenated sequence analysis gives better resolution in sequence similarity search and phylogenetic analysis.

**Please revise this as**

 Concatenated sequences provide much better resolution in sequence similarity search and phylogenetic analysis.

 The sequence accession numbers GU470907.1 and KF933785.1 classified as *S. mitis* showed a higher maximum and total alignment score with *S. oralis* than *S. mitis* ( Table 3).

**Please revise this as**

 Two sequences (accession numbers GU470907.1 and KF933785.1) from *S. mitis* had a higher maximum and total alignment score to sequences from *S. oralis* than *S. mitis* ( Table 3). PLEASE PROVIDE THE ACCESSION NUMBERS FROM S. ORALIS THAT PRODUCED THIS RESULT.

 Whereas the sequence (OM368574.1; classified as *S. mitis*) showed a higher sequence alignment score with *S. pneumoniae.*

**Please revise this as**

 Furthermore, yet another sequence from *S. mitis *(accession number OM368574.1) had a higher alignment score to sequences from *S. pneumoniae. *PLEASE PROVIDE THE ACCESSION NUMBERS FROM S. PNEUMONIAE THAT PRODUCED THIS RESULT.

Figure 3A shows a maximum likelihood tree of the nine 16S rRNA gene sequences with four concatenated species-specific reference libraries.

**Please revise this as**

 The maximum likelihood phylogenetic tree based on four concatenated species-specific reference libraries and the sequences of nine 16S rRNA genes is shown in Figure 3A. [THE LEGEND IS INSUFFICIENT; PLEASE PROVIDE MORE DETAILS IN THE LEGEND. ALSO, FIGURE 3B HAS NOT BEEN CALLED IN THE RESULTS SECTION, IT HAS BEEN CALLED DIRECTLY IN THE DISCUSSION SECTION]

 The concatenated GU470907.1 and KF933785.1 sequences showed a phylogenetic relationship with *S. oralis* and sequence OM368574.1 was genetically related to *S. pneumoniae.* 

**Please revise this as**

 [BASED ON THE ACCESSION NUMBERS, GU470907.1 and KF933785.1 CANNOT BE CONCATENATED SEQUENCES, PLEASE CHECK AND CORRECT]

 Two sequences (accession numbers GU470907.1 and KF933785.1) from *S. mitis* appeared to be phylogenetically closer to *S. oralis*, and yet another sequence from *S. mitis *(accession number OM368574.1) from *S. mitis* was closer to *S. pneumoniae.* [PLEASE NOTE: THE BOOTSTRAP VALUES ARE RATHER LOW; THEREFORE THE RESULTS NEED TO BE INTERPRETED CAREFULLY]

 These results indicate that the species-specific concatenated 16S rRNA reference libraries have great potential in the taxonomic classification.

**Please revise this as**

 These results further confirm that species-specific concatenated 16S rRNA reference libraries provide much better taxonomic resolution.

 Hence, the study suggests the usage of concatenated variable 16S rRNA copies for sequence similarity and phylogeny-based species identification.

**Please revise this as**

 Therefore, this study recommends using concatenated sequences of 16S rRNA genes for sequence similarity- and phylogeny-based species identification.

 A species-specific reference library with concatenated 16S rRNA gene copies provides better resolution in phylogenetic analysis than the single copy inference.

 THE SENTENCE ABOVE MAY BE DELETED BECAUSE A SIMILAR STATEMENT HAS ALREADY BEEN MADE.

Is the work clearly and accurately presented and does it cite the current literature?

Partly

If applicable, is the statistical analysis and its interpretation appropriate?

Not applicable

Are all the source data underlying the results available to ensure full reproducibility?

Yes

Is the study design appropriate and is the work technically sound?

Yes

Are the conclusions drawn adequately supported by the results?

Partly

Are sufficient details of methods and analysis provided to allow replication by others?

Yes

Reviewer Expertise:

Bacterial Genomics, Bacterial Taxonomy, Comparative Genomics, Pathogenomics

I confirm that I have read this submission and believe that I have an appropriate level of expertise to confirm that it is of an acceptable scientific standard, however I have significant reservations, as outlined above.

# Response

**Published:** 2023-03-22

**Authors:** Bobby Paul

**Affiliations:** Manipal University, India

Dear Sir,

 Thank you very much for your critical review and valuable suggestions for manuscript improvement. I have revised the manuscript by addressing all the suggestions and resubmitted it for your consideration. I sincerely hope that the modified manuscript is sufficiently improved, and kindly respond if you have any further questions or comments.

 Thank you

 Kind Regards

 Bobby Paul
